# Organic/Inorganic Hybrid Fibers: Controllable Architectures for Electrochemical Energy Applications

**DOI:** 10.1002/advs.202102859

**Published:** 2021-10-11

**Authors:** Fangzhou Zhang, Peter C. Sherrell, Wei Luo, Jun Chen, Wei Li, Jianping Yang, Meifang Zhu

**Affiliations:** ^1^ State Key Laboratory for Modification of Chemical Fibers and Polymer Materials College of Materials Science and Engineering Donghua University Shanghai 201620 P. R. China; ^2^ Department of Chemical Engineering The University of Melbourne Parkville VIC 3010 Australia; ^3^ ARC Centre of Excellence for Electromaterials Science Intelligent Polymer Research Institute (IPRI) Australian Institute of Innovative Materials (AIIM) University of Wollongong Wollongong NSW 2522 Australia; ^4^ Department of Chemistry Laboratory of Advanced Materials Shanghai Key Laboratory of Molecular Catalysis and Innovative Materials iChEM and State Key Laboratory of Molecular Engineering of Polymers Fudan University Shanghai 200433 P. R. China

**Keywords:** controllable architectures, electrochemical properties, energy storage and conversion, hybrid fibers, organic/inorganic hybrid

## Abstract

Organic/inorganic hybrid fibers (OIHFs) are intriguing materials, possessing an intrinsic high specific surface area and flexibility coupled to unique anisotropic properties, diverse chemical compositions, and controllable hybrid architectures. During the last decade, advanced OIHFs with exceptional properties for electrochemical energy applications, including possessing interconnected networks, abundant active sites, and short ion diffusion length have emerged. Here, a comprehensive overview of the controllable architectures and electrochemical energy applications of OIHFs is presented. After a brief introduction, the controllable construction of OIHFs is described in detail through precise tailoring of the overall, interior, and interface structures. Additionally, several important electrochemical energy applications including rechargeable batteries (lithium‐ion batteries, sodium‐ion batteries, and lithium–sulfur batteries), supercapacitors (sandwich‐shaped supercapacitors and fiber‐shaped supercapacitors), and electrocatalysts (oxygen reduction reaction, oxygen evolution reaction, and hydrogen evolution reaction) are presented. The current state of the field and challenges are discussed, and a vision of the future directions to exploit OIHFs for electrochemical energy devices is provided.

## Graphical abstract

Organic/inorganic hybrid fibers (OIHFs) are extensively investigated for electrochemical energy applications. This review summarizes the advances on OIHFs from their controllable structural design to electrochemical energy applications. Opportunities and remain key challenges for future research are highlighted.

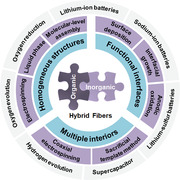

## 1 Introduction

**Figure 1:**
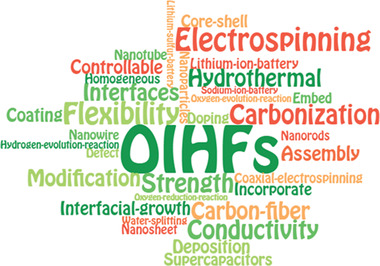
Keywords of OIHFs in the past ten years. (Image created by wordle. net.)

Organic/inorganic hybrid fibers (OIHFs) are a family of flexible pseudo‐1D materials, broadly possessing relatively high aspect ratio (>100, ø < 100 µm) and discrete organic/inorganic species domains that have attracted great attention for various electrochemical energy applications.^[^ ^1^, ^2^ ^]^ The broad family of materials with various chemistries of components, the ability to form hierarchical structures, and tunable and heterogenous electronic properties are inherently powerful for a wide range of applications, best presented by the diversity of keywords in OIHFs over the past 10 years (**Figure** **1**). OIHFs do not simply combine the intrinsic merits of individual entities, rather, well designed OIHFs generate advantageous new properties owing to synergistic interactions between of organic and inorganic components.^[^ ^3^, ^4^, ^5^, ^6^ ^]^ In general, an organic fiber matrix has large specific surface area, high flexibility, low density as well as unique anisotropic properties, which is considered to be an ideal substrate for incorporating various inorganic components.^[^ ^7^, ^8^, ^9^, ^10^ ^]^ Inorganic species such as heteroatoms, metal (or nonmetal) nanoparticles, and their compounds can be combined with the fiber matrix in different forms to endow this organic fiber matrix exceptional electrochemical properties.

The diversity of choice of organic/inorganic constituent species in OIHFs and their hybrid structures provides great possibility to tune and enhance their electrochemical properties. To date, a variety of synthetic approaches have been developed to fabricate functional OIHFs. By incorporating inorganic species into the organic fiber matrix during the fiber fabrication processes (electrospinning, liquid phase, etc.), the highly uniform OIHFs with controllable overall architectures are fabricated.^[^ ^11^, ^12^ ^]^ Of note, the assembly of organic and inorganic components at the molecular level represents a brand‐new horizon for fabricating OIHFs, which offers a powerful platform for accurately control the proportion and spatial location of organic/inorganic species. Additionally, OIHFs with multiple interiors are generally prepared by encapsulating inorganic component in the fiber matrix. This encapsulation process enables the morphology and spatial distribution of the inorganic components to be well controlled. Surface functionalization of the organic fibers can be realized by interface engineering strategies (surface deposition, dip‐coating, interfacial growth, etc.).^[^ ^13^, ^14^, ^15^, ^16^, ^17^ ^]^ The geometric configurations reported of inorganic components decorated on fiber surfaces include nanoparticles, nanodots, conformal layers, nanorods, nanowires, and nanosheets. The rich chemical and structural tailorability of hybrid architectures has stimulated extensive investigations on OIHFs for electrochemical energy applications.

**Figure 2:**
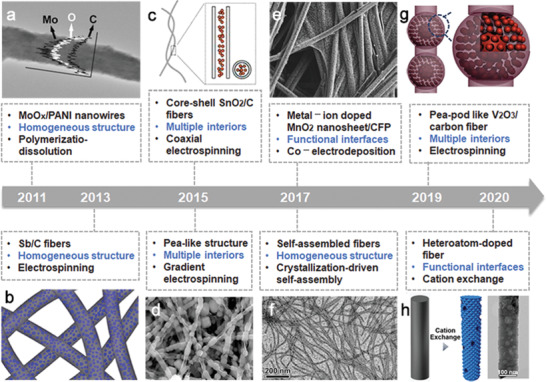
The representative architectures and timeline of OIHFs. a) Reproduced with permission.^[^ ^18^ ^]^ Copyright 2011, Wiley‐VCH. b) Reproduced with permission.^[^ ^19^ ^]^ Copyright 2013, American Chemical Society. c) Reproduced with permission.^[^ ^20^ ^]^ Copyright 2015, American Chemical Society. d) Reproduced with permission.^[^ ^21^ ^]^ Copyright 2015, Nature. e) Reproduced with permission.^[^ ^22^ ^]^ Copyright 2017, Wiley‐VCH. f) Reproduced with permission.^[^ ^23^ ^]^ Copyright 2017, Elsevier. g) Reproduced with permission.^[^ ^24^ ^]^ Copyright 2019, Elsevier. h) Reproduced with permission.^[^ ^25^ ^]^ Copyright 2020, Wiley‐VCH.

**Figure 3:**
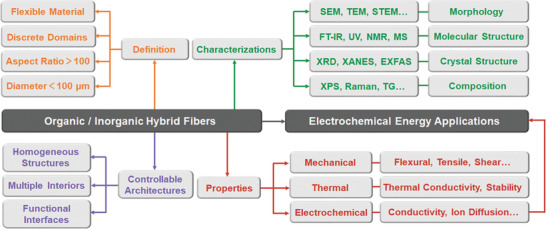
The schematic image illustrates the general aspects of OIHFs, which includes definition, characterizations, controllable architectures, and properties.

Significant efforts have been made to push OIHFs from laboratory research toward practical applications, and **Figure** **2** lists the developments of representative architectures surrounding OIHFs for electrochemical energy applications from 2011 to 2020.^[^ ^18^, ^19^, ^20^, ^21^, ^22^, ^23^, ^24^, ^25^ ^]^ While the fabrication of OIHFs have been well reviewed,^[^ ^26^, ^27^, ^28^, ^29^, ^30^, ^31^ ^]^ the relationship between their advanced synthetic strategies, tuneable structural features, and their resulting electrochemical performance have not been assessed in detail. In this review, we present a comprehensive overview of the controllable architectures and electrochemical energy applications of OIHFs (**Figure** **3**). After a brief introduction, the controllable construction of OIHFs is described in detail through precise tailoring of the overall, interior, and interface structures. Afterward, several important electrochemical energy applications including rechargeable batteries, supercapacitors, and electrocatalysts are presented. Finally, we end this review with a perspective on remaining challenges and future research directions of OIHFs for electrochemical energy applications.

## 2 Brief Overview of OIHFs

### 2.1 Characteristics

Although OIHFs have made great progress in recent years, the definition of this type of material is still ambiguous. Herein, based on the morphology and composition of the host material, we define OIHFs as a flexible material with aspect ratio of more than 100 and diameter of less than 100 µm which possess discrete domains of organic and inorganic components. The overall properties of OIHFs do not simply result from the sum of the individual species, but also from the strong synergy between components that occurs at the organic/inorganic interface. The interaction between organic and inorganic components, via covalent bonds, ionic bonds, hydrogen bonds, van der Waals forces, and electrostatic interactions, plays a major role in defining the physicochemical properties of hybrid fibers.

OIHFs are commonly produced from polymer fiber precursors including poly vinyl alcohol (PVA), polyacrylonitrile (PAN), polyamide, polyimide, polyurethane (PU), and conducting polymer fibers, such as polypyrrole (PPy), polyaniline (PANI), and poly(3,4‐ethylenedioxythiophene) (PEDOT). These 1D organic components are coupled to a wide variety of inorganic materials including precious noble metals (e.g., gold (Au), silver (Ag), platinum (Pt), and palladium (Pd)); nonprecious metals (e.g., iron (Fe), cobalt (Co), nickel (Ni), copper (Cu), zinc (Zn), titanium (Ti), vanadium (V), molybdenum (Mo), tungsten (W), tin (Sn), and antimony (Sb)), along with their compounds (e.g., oxides, carbides, nitrides, sulfides, phosphides, and carbonitrides); nonmetallic elements (e.g., aluminum (Al), silicon (Si)) as well as their oxides; and heteroatoms (e.g., nitrogen (N) boron (B), phosphorus (P), and sulfur (S)).

The mechanical properties of OIHFs (e.g., flexural strength, tensile strength, shear strength, storage modulus, Young's modulus, and toughness) are the basic aspects that need to be considered when employing such fibers in electrochemical energy devices. These mechanical properties are primarily controlled by the flexibility, aspect ratio, and density of the organic fiber matrix.^[^ ^32^ ^]^ A leading example of these mechanical properties was presented by Tang and co‐workers, where soft nylon fabric with high elasticity, toughness, and low density was used as the flexible substrate to deposit uniform Cu–Ni and amorphous silicon layer.^[^ ^33^ ^]^ The as‐prepared electrode exhibits high flexibility and mechanical stability over 50 000 bends. In addition, inorganic components could also affect the mechanical properties of OIHFs. Yadav et al. explored the static and dynamic mechanical properties of a hybrid multiscaled Ni‐coated CF–epoxy composite.^[^ ^34^ ^]^ They found that the uniformity and particle size of surface Ni coating affect the mechanical properties to a certain extent. The Ni‐coated CF composites showed a 69% improvement in interlaminar shear strength and the flexural strength reaches a maximum of 330 MPa.

The high electrical conductivity of OIHFs is necessary for use in electrochemical energy devices.^[^ ^35^, ^36^ ^]^ Improvement of the electrical conductivity of OIHFs can be realized by using conducting polymers or by varying the carbonization process of the polymer fibers listed above. The fabrication of polymer‐based carbon fibers mainly includes two processes: polymer fibers formation and subsequent carbonization. These carbon fibers not only play a preponderant role in modulating electrical conductivity and mechanical strength, but also provide an ideal template for the composite of inorganic components. Introducing inorganic materials such as metals to carbon fiber matrix can further improve the electrical conductivity, and subsequently their electrochemical properties. Ma et al. reported a highly conductive stretchable Ag–PU fibers, which provided high conductivity (41 245 S cm^−1^) as well as high Young's modulus (731.5 MPa) and ultimate strength (39.6 MPa).^[^ ^37^ ^]^ Furthermore, the carbon fiber could efficiently decrease the electrical contact resistance between adjacent metal nanoparticles, thereby providing good electrical contact during the electrochemical reactions. These characteristics pave the way for the applications of OIHFs in multiple electrochemical research fields.

### 2.2 Characterization Techniques

To uncover the relationship between chemical and physical structural features and properties, multiple advanced characterization techniques have been applied to reveal the nature of OIHFs. Due to the interdisciplinary nature of OIHFs, their structural analysis generally requires a combination of the characterization techniques of organic and inorganic materials. It is expected that a deeper comprehension of the characterization techniques will facilitate the advanced structural design of functional OIHFs.

The molecular structure and chemical composition of polymer fiber can be determined by four major spectroscopic methods, namely, Fourier transform‐infrared (FT‐IR), Raman spectroscopy, UV–vis spectroscopy, nuclear magnetic resonance, and mass spectrum. Thermal analysis methods such as differential scanning calorimeter, dynamic thermomechanical analysis, and thermogravimetric analysis (TGA) are used to analyze the dependence of the physical properties of polymer fiber on temperature. Remarkably, FT‐IR technique plays a crucial role in detecting the bonding state between organic and inorganic species. For instance, Qiao and co‐workers found that the characteristic stretching modes of CN heterocycles in PCN‐CFP (phosphorus‐doped g‐C_3_N_4_ grown on carbon fiber paper) show apparent shifts as compared to pure g‐C_3_N_4_, indicating strong interaction between in situ grown phosphorus‐doped g‐C_3_N_4_ and carbon fiber paper.^[^ ^38^ ^]^ Furthermore, X‐ray photoelectron spectroscopy (XPS) is able to investigate the elemental valence state in surface of OIHFs. In a work of Fang and co‐workers, the observed Co—N and Fe—N bonds in Co 2p and Fe 2p spectrums indicate the strong interaction between FeCo nanoparticles and N‐doped carbon fibers.^[^ ^39^ ^]^ Besides, X‐ray absorption near edge structure and extended X‐ray absorption fine structure also provide information about the electronic state of surface inorganic components (especially single metal atoms).

To determine the macrostructure of OIHFs, the phase composition and content of organic/inorganic components should be deeply investigated. X‐ray diffraction is widely used to probe the crystal structure and phase purity of hybrid fibers, the position of the diffraction peak represents the chemical composition of the crystal phase. Besides, the content of organic and inorganic species in OIHFs is another critical factor affecting the structural features. Generally, TGA can probe the content of carbon fiber through weight loss, inductive coupled plasma emission spectrometer is used to determine the content of trace inorganic components, and XPS could detect the atomic ratio of each element on the fiber surface. Furthermore, the graphitization degree of carbon fibers could be acquired from the intensity of disordered (D) and graphite (G) bands from Raman spectra. For example, Zhang and co‐workers found that the *I*_D_/*I*_G_ ratio decreases from 1.202 to 1.088 as the FeCl_3_·6H_2_O concentration increases, indicating that an appropriate amount of inorganic precursor helps to produce carbon fibers with a higher graphitization degree.^[^ ^40^ ^]^ For porous materials, the specific surface area and pore size distribution can be detected by N_2_ adsorption–desorption isotherms and BJH pore‐size distributions, respectively. According to the Brunauer–Emmett–Teller (BET) method, the specific surface area and pore‐size distribution (both of which are critical for electrochemical performance) of OIHFs can be calculated.

Based on their hierarchical structure, with features ranging from the macroscale to the nanoscale, the morphology of OIHFs can be investigated by the digital photography, optical microscopy, atomic force microscopy, scanning electron microscopy (SEM), and transmission electron microscopy (TEM). The spatial chemical distribution of OIHFs is analyzed on the micro‐to‐nanoscale by energy‐dispersive X‐ray spectroscopy and high angle annular dark‐field scanning transmission electron microscopy. In addition, some advanced microscopy techniques, such as spherical aberration‐corrected transmission electron microscopy and scanning tunneling microscope, are applied to reveal the structural nature and coordination environment of OIHFs at the atomic level.

## 3 Controllable Architectures of OIHFs

**Figure 4:**
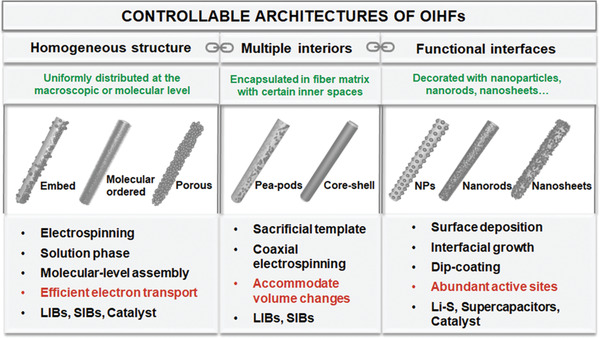
Controllable architectures and key points of OIHFs.

The ability to spatially control both chemical distributions and hierarchical physical structure is a critical advantage of OIHFs for electrochemical energy applications. According to the spatial distribution of organic and inorganic components, these OIHFs can be categorized into three groups, OIHFs with: homogeneous structures; multiple interiors; and functional interfaces (**Figure** **4**). This section will focus on the construction of OIHFs with controllable architectures as well as the relationship between structure feature and properties.

### 3.1 OIHFs with Homogeneous Structures

Homogeneous structures of OIHFs mean that the organic and inorganic components are uniformly distributed at the macroscopic or molecular level. This subsection lists several representative strategies for preparing OIHFs with homogeneous structures, including electrospinning, liquid phase, and molecular‐level assembly.

Electrospinning is a facile and versatile approach to produce homogeneous fibers with controlled overall properties.^[^ ^41^, ^42^, ^43^, ^44^ ^]^ OIHFs with different textural characteristics can be easily prepared by controlling organic/inorganic precursors and electrospinning conditions.^[^ ^45^, ^46^, ^47^, ^48^ ^]^ The synthesis of OIHFs through electrospinning can be roughly divided into two types. One is based on the electrospinning of inorganic precursor and polymer solution, sometimes followed by postprocessing such as calcination. The other is to directly embed the prepared inorganic particles in the fiber matrix. Shao et al. first reported that a PVA/silica composite with different silica content can be prepared via the electrospinning technique by using the PVA and TEOS mixture as precursor.^[^ ^49^ ^]^ By simply controlling the amount of TEOS precursor, PVA/silica fibers with different silica content can be obtained. After this achievement, by electrospinning a mixed solution containing titanium precursor and surfactant P123, and subsequent heat treatment at a suitable temperature to selectively remove the organic surfactant, mesoporous TiO_2_ hollow fibers with high specific surface area were also produced.^[^ ^50^ ^]^

**Figure 5:**
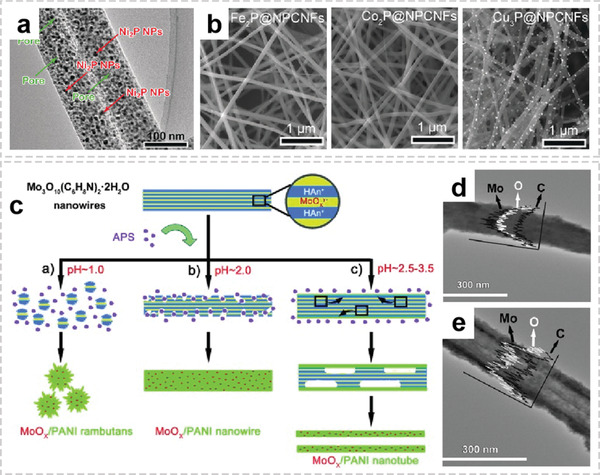
Representative architectures of OIHFs with homogenous structures. a) TEM image of as‐prepared Ni_2_P@NPCNFs. b) FESEM imagesof M*_x_* P@NPCNFs (M = Fe, Co, Cu). a,b) Reproduced with permission.^[^ ^56^ ^]^ Copyright 2017, Wiley‐VCH. c) The schematic illustration for the formation of MoO*_x_*/PANI nanocomposites. FETEM image of the X‐ray profile for Mo, C, and O domains in the d) nanowire and e) nanotube. c,d) Reproduced with permission.^[^ ^18^ ^]^ Copyright 2011, Wiley‐VCH.

Metal or their compounds modified organic fibers can be produced by electrospinning the mixed solution of polymer and metal salt precursor and subsequent calcination.^[^ ^51^, ^52^, ^53^, ^54^, ^55^ ^]^ Bao and co‐workers reported an electrospinning‐based reduction approach to in situ embedded ultrafine nickel phosphides in N‐doped porous carbon nanofibers (NPCNFs) (**Figure** **5**a).^[^ ^56^ ^]^ This facile approach is also applicable to other metals such as Fe_2_P, Co_2_P, and Cu_3_P. As shown in Figure 5b, these M*_x_* P nanoparticles were evenly embedded in the carbon NFs matrix, and the fibrous structure was well‐maintained. In addition to adding inorganic precursors to the spinning solution, Zhang et al. selected a series of as prepared metal–organic frameworks (MOFs) and embedded them into different polymers to fabricate functional hybrid nanofibers.^[^ ^57^ ^]^ They found that polymers such as PAN, PS, and PVP can also be used as carrier polymers, and the as‐obtained hybrid fibers shows high flexibility even under high MOF content.

Liquid phase synthesis is an efficient approach for synthesizing hybrid nanowire or nanofiber because of its various advantages such as high reactivity of reactants, easy control of solution reactions, and low energy consumption.^[^ ^58^ ^]^ The Tang group reported a series of novel organic/inorganic hybrid nanostructures using liquid phase synthesis, which are of great significance for the controllable synthesis of sub‐nanometer periodic hybrid structures. For example, novel hybrid GeO*_x_*/ethylenediamine (EDA) nanowires were prepared, in which EDA connects the inorganic units through (N—H···O—Ge) H‐bonding to form a unique organic/inorganic hybrid nanostructure.^[^ ^59^ ^]^ The GeOx/EDA nanowires were synthesized by directly mixing GeO_2_ with Fe_2_O_3_ and then treating the mixture in EDA solution at 200 °C for 5 days. They proposed a Fe_2_O_3_‐assisted growth mechanism, where the H‐bonding generated from assistance of Fe_2_O_3_ induced the anisotropic growth of hybrid nanowires. After this achievement, this group further proposed a facile oxidative polymerization method to controllable convert the precursor of Mo_3_O_10_(C_6_H_8_N)_2_·2H_2_O nanowires into organic/inorganic hybrid MoO*_x_*/PANI nanostructures.^[^ ^18^ ^]^ By simply adjusting the pH value of reaction solution, the hybrid nanostructures with different morphologies such as rambutan‐like particles, nanowires, and nanotubes can be obtained (Figure 5c–e).

**Figure 6:**
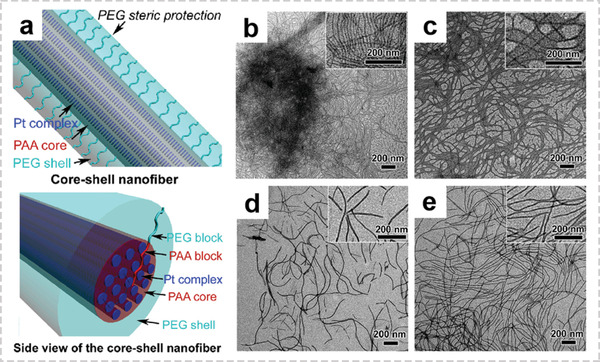
Representative architectures of OIHFs with homogenous structures. a) Schematic illustration of the core‐shell nanofiber. TEM images of the nanofibers formed by complex 1+PEG_45_‐*b*‐PAA_30_ b) and complex 1+PEG_45_‐*b*‐PAA_45_ c) in a mixed solvent of acetonitrile–methanol–water (1:1:8, v/v/v), respectively. TEM images of the nanofibers prepared from the mixtures with complex/carboxylic acid molar ratios of 0.06/1 d) and 0.12/1 e). Reproduced with permission.^[^ ^23^ ^]^ Copyright 2017, Elsevier.

Recently, molecular‐level assembly of organic and inorganic components has attracted much attention. This approach offers a powerful platform for accurately control the proportion and spatial distribution of organic/inorganic species. The noncovalently connected micelle (NCCM) strategy, which directly guides the formation of fibrous nanostructures by using pairs of complementary polymers as building blocks, have been extensively studied.^[^ ^60^ ^]^ Zhang et al. synthesized 1D crystalline fibrous nanostructures of platinum(II) complexes through supramolecular coassembly of platinum(II) complexes with PEG‐*b*‐PAA diblock copolymers (**Figure** **6**a).^[^ ^23^ ^]^ The PAA block in PEG‐*b*‐PAA could interact with the platinum(II) complex through electrostatic interaction and trigger the assembly of platinum(II) complexes inside the core of the polymer fibers. TEM images shown in Figure 6b–e indicate that the diameters of the nanofibers increased with the PAA block length in PEG‐*b*‐PAA, and the lengths of the nanofibers increased with the complex/polymer feed ratios. This fibrous nanostructure with controlled length, diameters, and compositions shows great flexibility in tailoring overall properties. Recently, Tian et al. prepared a block copolymer with a crystallizable core‐forming block and functional corona‐forming segment through the living crystallization‐driven self‐assembly seeded growth method.^[^ ^61^ ^]^ By controlling the mass ratio of the two block copolymer containing catalyst and photosensitizer, the spatial location of functional blocks can be easily tuned. The homogenous distribution of materials in this class of OIHFs is ideal for consistent and reproducible electrochemical energy storage and conversion devices.

### 3.2 OIHFs with Multiple Interiors

OIHFs with multiple interiors refer to inorganic component are encapsulated in the fiber matrix, and the morphology and surrounding spaces of the inorganic components can be well controlled. Internal void spaces in the fiber matrix can accommodate the volume changes associated with electrochemical reactions, thus limiting structure degradation when these OIHFs are used as electrode materials. This subsection discusses several representative strategies for preparing OIHFs with multiple interiors.

**Figure 7:**
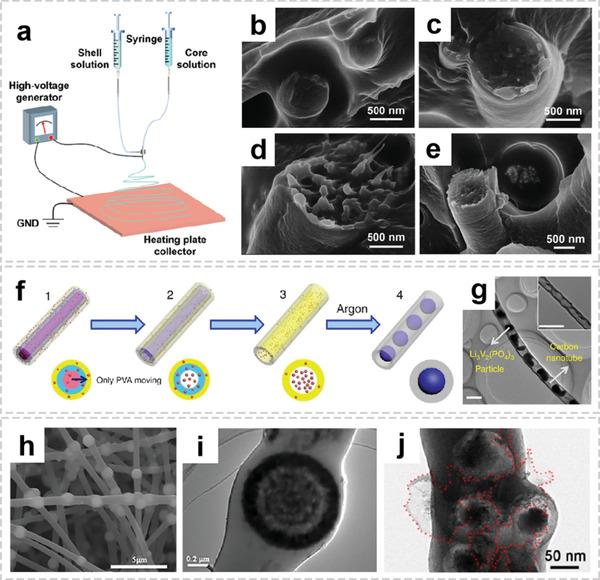
Representative architectures of OIHFs with multiple interiors. a) Coaxial electrospinning apparatus. b–e) Cross‐section of electrospun PDMS/BT@PVDF nanofibers with the BT NP contents of 0.3, 1.2, 2.4, and 3.6 wt%. a‐e) Reproduced with permission.^[^ ^64^ ^]^ Copyright 2020, Elsevier. f) Preparation process of pea‐like nanotubes. g) TEM images of carbon nanotubes after removing Li_3_V_2_(PO_4_)_3_ with hydrogen fluoride with scale bar at 500 nm. f,g) Reproduced with permission.^[^ ^21^ ^]^ Copyright 2015, Nature. h) SEM and i) TEM images of the V_2_O_3_@NSCNFs. h,i) Reproduced with permission.^[^ ^24^ ^]^ Copyright 2019, Elsevier. j) TEM of the 3D FSiGCNF film. The Si NPs possessing off‐center hollow spheres embed in the graphene/carbon matrix. Reproduced with permission.^[^ ^65^ ^]^ Copyright 2016, American Chemical Society.

Coaxial electrospinning is a direct approach to synthesize OIHFs with more diverse interior morphologies.^[^ ^62^ ^]^ The overall functionalities can be well tailored due to the coaxially compositing of diverse organic/inorganic components in radial direction.^[^ ^63^ ^]^ Zhu and co‐workers used PVDF as the outer layer and polydimethylsiloxane/barium titanate nanoparticles (PDMS/BT) as the core to fabricate PDMS/BT@PVDF core–shell nanofibers through modified two‐component coaxial electrospinning (**Figure** **7**a).^[^ ^64^ ^]^ Figure 7b–e shows the cross‐section FE‐SEM images of PDMS/BT@PVDF coaxial nanofibers with different BT NP concentrations. It can be observed that the BT NPs tend to aggregate into bigger clusters when BT NP content increase, suggesting that the dispersion degree is greatly reduced. Although coaxial electrospinning has demonstrated its ability to manufacture delicate structures, it has the limitations with low productivity and complex preparation procedures. In order to enhance the yield and repeatability, the Mai group proposed a universal gradient electrospinning and followed by controlled pyrolysis to synthesize a series of pea‐like fibrous nanostructures.^[^ ^21^ ^]^ As shown in Figure 7f, the low‐, middle‐, and high‐molecular‐weight PVA and inorganic precursors are first electrospun into homogeneous fibers. As the pyrolysis temperature increased, the PVA decomposes and quickly moves toward the outer high‐weight PVA layer, leaving inorganic materials in the center of the fiber. After annealing at high temperature under argon, the pea‐like hybrid fibers are obtained (Figure 7g). It is proved that this method is suitable for the synthesis of various metal‐based pea‐like nanostructures.

Inorganic materials with designed architectures show greater flexibility in regulating their composition and structures, and have been used in the synthesis of OIHFs with multiple interiors. Gou et al. successfully synthesized yolk–shell structured V_2_O_3_ microspheres and embedded them into N, S codoped carbon fibers.^[^ ^24^ ^]^ According to Figure 7h,i, the V_2_O_3_ microspheres maintain their original yolk–shell structure after carbonization and sulfur doping process, forming a pea‐pod like structure. The sacrificial template method offers a convenient route for fabricating OIHFs with well‐defined internal voids. Zhu et al. prepared the Si/NiO/graphene/PAN nanofiber web through electrospinning, and NiO was used as a sacrificial template to construct void space inside the fiber (Figure 7j).^[^ ^65^ ^]^ Further, Ma et al. utilized the sulfur nanoparticles with flowable and removable characteristics as a template to prepare porous SnO_2−_ *_x_*/C nanofibers.^[^ ^66^ ^]^ This accurate and controllable built‐in void space could effectively accommodate volume expansion, which has been widely used in electrochemical energy storage systems.

### 3.3 OIHFs with Functional Interfaces

OIHFs with functional interfaces refer to inorganic components with diverse chemical composition, size, and morphology distributed on the fiber surface. For electrode materials in electrocatalysts, accessible active sites, and electronic‐diffusion interfaces are highly desired. In this subsection, we focus primarily on controllable interface modification approaches of OIHF, including surface deposition, interfacial growth, and other new methods.

Surface deposition methods enable the deposition of uniform inorganic coatings on a fibrous matrix, and the thickness and uniformity of the coating materials can be precisely controlled.^[^ ^67^ ^]^

**Figure 8:**
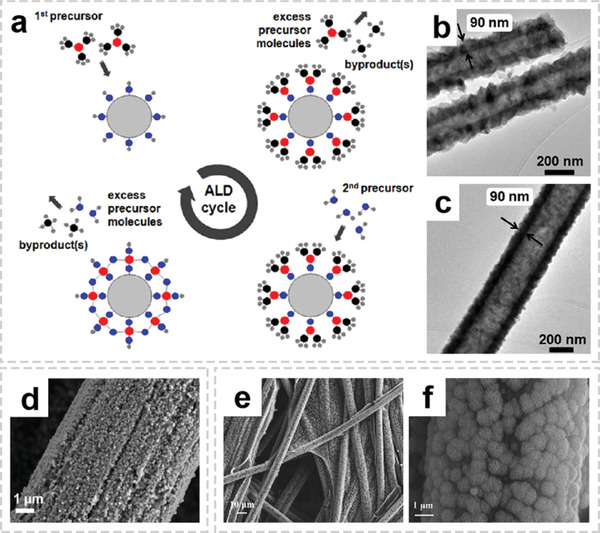
Representative architectures of OIHFs with functional interfaces. a) Schematic representations of the ALD process for the production of core–shell polymer–inorganic nanofibers. TEM images of b) 8%‐nylon 6,6/FA‐ZnO and c) 5%‐nylon 6,6/HFIP‐ZnO core–shell nanofibers. a‐c) Reproduced with permission.^[^ ^70^ ^]^ Copyright 2012, American Chemical Society. d) SEM image of CoP/CFP‐H. Reproduced with permission.^[^ ^76^ ^]^ Copyright 2018, American Chemical Society. e,f) FESEM images of the metal‐ion‐doped MnO_2_ ultrathin nanosheet/CFP composite electrode with different magnifications. e,f) Reproduced with permission.^[^ ^22^ ^]^ Copyright 2017, Wiley‐VCH.

Atomic layer deposition (ALD) is a thin film technology, which allows metal (oxides) to be deposited on the surface of the fiber matrix in a conformal and uniform manner.^[^ ^68^, ^69^ ^]^ For instance, Kayaci et al. successfully synthesized polymer–inorganic core–shell nylon 6,6‐ZnO nanofiber by combining of electrospinning and ALD processes (**Figure** **8**a).^[^ ^70^ ^]^ They found that the ZnO shell layer with uniform thickness throughout the porous fibrous structure can be deposited regardless of the fiber diameter variation (Figure 8b,c). Several other metal (oxides), such as W, Pt, TiO_2_, CoO*_x_*, and Al_2_O_3_ could also grow a conformal coating on the fiber substrate by ALD.^[^ ^71^, ^72^, ^73^, ^74^, ^75^ ^]^

Chemical vapor deposition (CVD) has been developed extensively for the preparation of metal nitrides, phosphides, and sulfides by postprocessing metal‐based compounds. Yu and Chua successfully fabricated CoP particles on 3D carbon fiber paper by a hydrothermal process and subsequent CVD phosphorization.^[^ ^76^ ^]^ As shown in Figure 8d, the crystalline Co_3_O_4_ nanocube particles on carbon paper are phosphorized into catalytically active CoP with a rounded and rough morphology.

Electrochemical deposition (ED) is a widely used technology that produces a thin coherent coating by flowing current through a circuit in a solution with two electrodes.^[^ ^77^, ^78^, ^79^ ^]^ Electrodeposition parameters including current density/voltage, additives, and temperature provide opportunities to adjust deposition characteristics of the deposited materials. Ye et al. prepared a metal‐ion (Fe, V, Co, and Ni)‐doped MnO_2_ ultrathin nanosheet/CFP composite using a facile anodic co‐electrodeposition method (Figure 8e,f).^[^ ^22^ ^]^ They confirmed that when the preparation parameter (i.e., the H_2_SO_4_ concentration of the electrolyte, the deposition current density and the electrolyte composition) changes, the morphology of the deposited layer will change accordingly.

**Figure 9:**
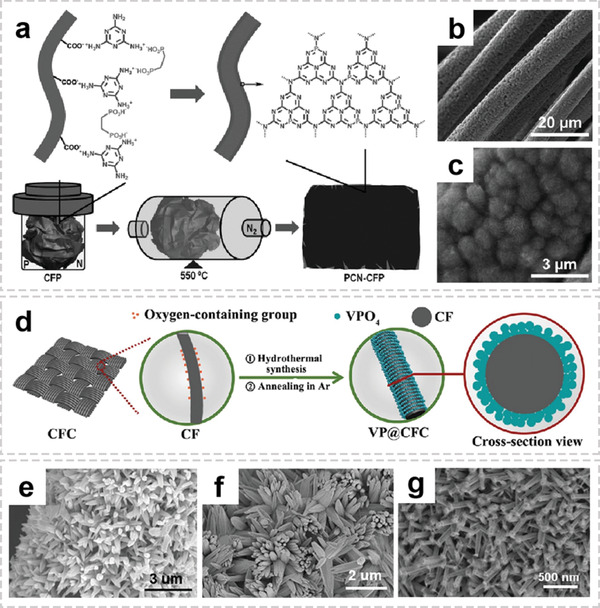
Representative architectures of OIHFs with functional interfaces. a) Fabrication of P‐g‐C_3_N_4_ nanostructures directly grown on CFP. b,c) SEM images of PCN‐CFP. a‐c) Reproduced with permission.^[^ ^38^ ^]^ Copyright 2015, Wiley‐VCH. d) Schematic illustration of the preparation of composite VP@CFC. Reproduced with permission.^[^ ^86^ ^]^ Copyright 2020, Elsevier. SEM images of e) CC@ZnO‐1, f) CC@ZnO‐2, and g) CC@ZnO‐3. (The metal sources are Zn(NO_3_)_2_·6H_2_O, ZnCl_2_·6H_2_O, and Zn(CH_3_COO)_2_, respectively.) e‐g) Reproduced with permission.^[^ ^87^ ^]^ Copyright 2019, Wiley‐VCH.

Interfacial growth refers to in situ grow functional inorganic materials on prepared fiber surface. By controlling the precursor and reaction conditions, coating materials with diverse geometry configurations (e.g., nanoparticles,^[^ ^39^ ^]^ nanorods,^[^ ^17^, ^80^ ^]^ nanosheet,^[^ ^81^, ^82^ ^]^ and nanotube^[^ ^83^ ^]^) can be facilely fabricated. In order to efficiently control the interfacial growth process of inorganic components, controlling interfacial interactions between the solution and the carbon fibers, often by functionalization process of the fiber matrix, is critical. Generally, carbon fibers can be functionalized through thermal, plasma treatment and acid oxidation to introduce abundant functional groups (such as carboxylic groups, hydroxyl groups) on the surface, which plays an important role as nucleation sites for the inorganic crystal seeds.^[^ ^84^ ^]^ PAN and PANI fibers with abundant amino and hydroxyl groups on the surface are also regarded as the ideal growth substrates.^[^ ^85^ ^]^ For example, Qiao group functionalized the commercial carbon fiber paper by oxidation treatment to make the surface rich in functional groups (e.g., —COO^−^), thereby enhance the interaction with basic melamine as the N source (**Figure** **9**a–c).^[^ ^38^ ^]^ In fact, in most cases, as long as the solution phase containing the inorganic precursors could wet the surface of an arbitrary conductive substrate, conformal coatings with good electronic connections between inorganic and organic components could be achieved. Furthermore, the composition of inorganic precursors as well as additive has an important influence on the morphology of inorganic components. Zhao and co‐workers prepared a self‐supported electrode with spherical VPO_4_ particles grown on carbon fiber cloth (VP@CFC) by hydrothermal synthesis method (Figure 9d).^[^ ^86^ ^]^ As the number of precursors added in the synthesis system increases, more and more VPO_4_ spheres grow on the CF surface, which further proves that controlling the concentration of precursors could control the number of particles on the CFC. Wang et al. explored the effects of different zinc sources on the morphology of ZnO array in CC@ZnO composites.^[^ ^87^ ^]^ ZnO is a typical polar crystal and anions have an important influence on its crystal morphology. When the anion in the reaction system is NO_3_^−^, Cl^−^, and CH_3_COO^−^, the corresponding ZnO microcrystal exhibited single‐like, clusters‐like, and tetrapod‐shaped morphology, respectively (Figure 9e–g). In another case, three different Zn–Co–S nanoarchitectures (nanosheets, nanoplates, and nanoneedles) grown on conductive carbon fiber paper were synthesized by tailoring the amount of urea and NH_4_F additive in solution.^[^ ^88^ ^]^ Bae et al. successfully grown uniform‐sized granular Ni–Co nanowires on carbon fibers by a hydrothermal method.^[^ ^89^ ^]^ They confirmed that cationic surfactant cetyltrimethylammonium bromide (CTAB) prefers to adhesion to the preferential faces of Ni–Co precursor seeds. As a result, the CTAB‐adsorbed faces are inactive, which is conducive to preferential growth of other surfaces.

**Figure 10:**
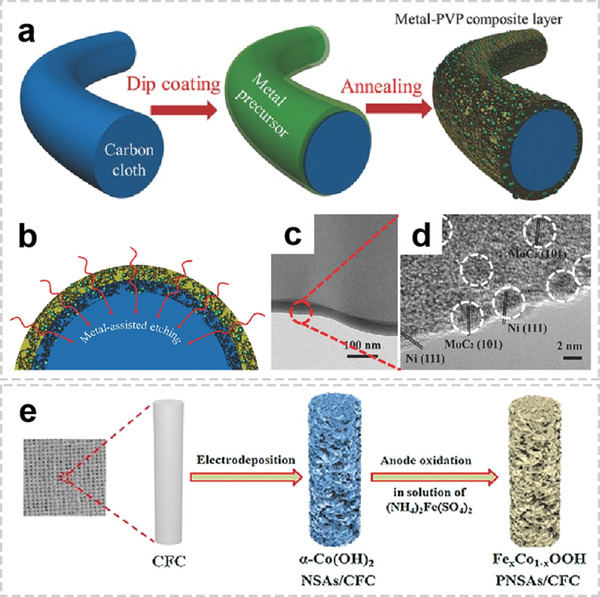
Representative architectures of OIHFs with functional interfaces. a) Fabrication process of metal‐based nanoparticles/N‐doped porous carbon hybrid catalysts. b) Cross‐sectional view of the interface. c) Low‐ and d) high‐magnification TEM images of NiMo‐PVP. a‐d) Reproduced with permission.^[^ ^9^ ^]^ Copyright 2017, Wiley‐VCH. e) Illustration of the fabrication of Fe*_x_* Co_1‐_ *_x_* OOH PNSAs/CFC. Reproduced with permission.^[^ ^93^ ^]^ Copyright 2018, Wiley‐VCH.

In addition to common interface modification methods mentioned above, some new methods are used in the synthesis of OIHFs. Dip‐coating enables the fabrication of homogeneous films with a high degree of control over the structure and thickness on various substrates.^[^ ^90^, ^91^ ^]^ In principle, the homogeneous metal precursor film can be deposit on the fiber matrix using metal salts as the precursor solution, and followed solvent evaporation leads to the formation of a solid metal (oxide) films. Zhang et al. successfully prepared a series of ultrafine transition metal‐based nanoparticles (Ni–Fe, Ni–Mo) embedded in N‐doped carbon cloth via dip‐coating method (**Figure** **10**a,b).^[^ ^9^ ^]^ First, the metal salts precursors uniformly covered on carbon fibers by dip‐coating, are then reduced into nanosized particles. The thermal reduction catalyzes an etching process of the carbon surface, which simultaneously induce porosity and nitrogen doping in the carbon fiber surfaces (Figure 10c,d). Furthermore, with a short reaction time and tunability, carbothermal shock method is an effective method for loading metal nanoparticles on carbon fibers. Hu and co‐workers controllably incorporated multiple immiscible elements into single fibers, which enables uniform nanoscale dispersions and high‐entropy mixing.^[^ ^92^ ^]^ The lower carbonization temperature leads to higher surface defect concentration in the carbon fiber, further resulting in smaller and more uniform particle dispersions. The catalytic activities of the metal elements also play an important role in nanoparticle size distribution. The metal species (Au and Pt) with higher catalytic activity exhibits smaller nanoparticles and more uniform distributions. Moreover, in order to further functionalized the prepared OIHFs, various post‐treatment methods like nitridation (sulfuration and phosphorization), plasma treatment, anodic oxidation, acid etching, and coating have also been employed. For example, Fe‐substituted CoOOH porous nanosheet arrays grown on carbon fiber cloth were synthesized by in situ anodic oxidation of *α*‐Co(OH)_2_ in (NH_4_)Fe(SO_4_)_2_ solution (Figure 10e).^[^ ^93^ ^]^ During the in situ transformation process, the partial CoO_6_ octahedral structure is exchanged by FeO_6_ octahedrons, which possesses higher electrocatalytic active sites.

### 3.4 Summary

In summary, multifarious OIHFs with homogeneous structures, multiple interiors, and functional interfaces can be successfully synthesized using various fabrication methods. Among these, electrospinning is the most versatile and convenient method to obtain OIHFs with controlled homogeneous or interior structures. Molecular‐level assembly offers a powerful platform for accurately control the proportion and spatial location of organic/inorganic species. Surface deposition and dip‐coating are considered the thin film technology to deposit uniform and dense coatings on a complex‐shaped fiber matrix. Interfacial growth possess the advantage of easily control the interface growth process and the morphology of inorganic components. OIHFs with well‐designed architectures could meet the needs of a broad range of electrochemical energy applications. The following section will describe these applications in detail.

## 4 Applications of OIHFs

Due to the large specific surface area, abundant active sites, and short ion diffusion length, OIHFs are considered to be excellent candidates for various electrochemical energy applications. This part elaborates the state‐of‐the‐art on OIHFs, from controllable synthesis to their successful applications in different types of electrochemical energy devices, including rechargeable batteries, supercapacitors, and electrocatalysts. More attention will be focused on the relationship between the structural feature and their electrochemical performance.

### 4.1 Rechargeable Batteries

The rapid growth of portable devices and electric vehicles (EVs), has brought increasing demands for high energy density batteries.^[^ ^94^ ^]^ The following subsection will discuss latest developments in OIHFs for rechargeable batteries, including lithium‐ion batteries (LIBs), sodium‐ion batteries (SIBs), and lithium–sulfur batteries (Li–S). Some representative electrode materials and corresponding electrochemical performance are listed in **Table** **1**.

**Table 1:** Performance comparison of different OIHFs electrode for LIBs/SIBs/Li–S applications

Application	Materials	Structure	Synthesis technique	Current density [mA g^−1^]	Cycle number	Capacity [mA h g^−1^]	Ref.
LIBs	*α*‐Fe_2_O_3_/CNFs	Homo‐	Electrospinning	50	75	488	^[^ ^40^ ^]^
	Fe_2_O_3_–C composite NFs	Homo‐	Electrospinning	1000	300	812	^[^ ^100^ ^]^
	SnO_2_–NiO/CNF	Homo‐	Electrospinning	50	100	448	^[^ ^101^ ^]^
	Ni/NiO/MnO_x_/CF	Homo‐	Electrospinning	100	200	1360	^[^ ^102^ ^]^
	SiO_2_/Sb@CNF	Homo‐	Electrospinning	500	200	450	^[^ ^111^ ^]^
	Si/graphene/CFs	Multi‐	Chemical etching	700	1050	2002	^[^ ^65^ ^]^
	Si@void@C NFs	Multi‐	Chemical etching	500	100	1045	^[^ ^108^ ^]^
	Si@TiO_2‐_ *_x_*/CFs	Multi‐	Coaxial electrospinning	200	50	945	^[^ ^109^ ^]^
SIBs	N‐CNF	Homo‐	Electrospinning	100	100	377	^[^ ^121^ ^]^
	TiO_2_/C NFs	Homo‐	Electrospinning	2000	1000	302	^[^ ^122^ ^]^
	Sn nanodots@PNC	Homo‐	Electrospinning	2000	1300	483	^[^ ^134^ ^]^
	C@Sb NFs	Homo‐	Electrospinning	1000	300	438	^[^ ^137^ ^]^
	SnO_2‐_ *_x_*/CFs	Multi‐	Sulfur dissolution	100	300	634	^[^ ^66^ ^]^
	PCNF@SnO_2_@C	Funct‐	ED	50	100	374	^[^ ^123^ ^]^
	MoS_2_@CF	Funct‐	Hydrothermal	80	100	184	^[^ ^129^ ^]^
	P/CFs@RGO	Funct‐	Vaporization condensation	1000	180	407	^[^ ^136^ ^]^
Li–S	TiN/C@S NFs	Homo‐	Electrospinning	335	300	685	^[^ ^149^ ^]^
	TiN‐VN@CNFs	Homo‐	Electrospinning	335	100	1110	^[^ ^151^ ^]^
	FeS@SPAN	Homo‐	Electrospinning	1000	500	689	^[^ ^152^ ^]^
	f‐CMWF	Funct‐	ALD	400	450	859	^[^ ^147^ ^]^
	Fe_3_O_4_‐NC@ACC	Funct‐	Impregnation	335	1000	780	^[^ ^148^ ^]^
	PCF/VN	Funct‐	Hydrothermal	167.5	250	1053	^[^ ^150^ ^]^
	MoS_2_@N‐CNFs	Funct‐	Hydrothermal	335	250	795	^[^ ^153^ ^]^
	SnS_2_@N‐CNFs	Funct‐	Hydrothermal	335	150	1010	^[^ ^154^ ^]^

#### 4.1.1 Lithium‐Ion Batteries

LIBs, with relatively high energy density as well as long cycle life, have been extensively applied in electric vehicles and consumer electronics.^[^ ^95^, ^96^ ^]^ These batteries typically have a carbonaceous anode (e.g., graphene), and an inorganic cathode (e.g., LiCoO_2_). However, the current mainstay electrode materials cannot meet the ever‐increasing demand of high energy density. Although many researches are devoted to improving the specific capacity of carbon fiber through structural modification, its capacity has approached the limit of carbonaceous materials.^[^ ^97^ ^]^ OIHFs have attracted great interest because of their intriguing electronic and physicochemical properties, and given their hybrid organic/inorganic nature have found potential applications as both anodes and cathodes in LIBs.^[^ ^98^ ^]^

**Figure 11:**
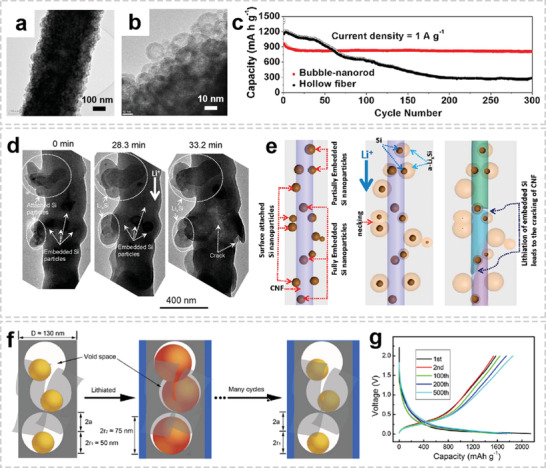
OIHFs for lithium‐ion batteries. a,b) TEM images of Fe_2_O_3_–C composite nanofibers. c) cycling performances of bare Fe_2_O_3_ hollow nanofibers and bubble‐nanorod‐structured Fe_2_O_3_–C composite nanofibers. a‐c) Reproduced with permission.^[^ ^100^ ^]^ Copyright 2015, American Chemical Society. d) Comparison of the lithiation characteristics of the particles attached to and embedded in the CNF. e) Schematic illustration of the lithiation characteristics of the particles attached to and embedded in the CNF. d,e) Reproduced with permission.^[^ ^107^ ^]^ Copyright 2012, American Chemical Society. f) Schematic diagram of the 3D FSiGCNF electrode design. g) Charge and discharge curves of the 3D FSiGCNF electrode obtained at a current density of 700 mA g^−1^ in the first, second, 100th, 200th, and 500th cycles. f,g) Reproduced with permission.^[^ ^65^ ^]^ Copyright 2016, American Chemical Society.

Compared to the graphite anode with a theoretical capacity of about 372 mA h g^−1^, conversion type anodes (e.g., metal oxides, metal sulfides, and phosphides) shows much higher specific capacity, which rely on the conversion reaction between lithium ions and the active materials.^[^ ^99^ ^]^ Dispersing various metal oxides into a fibrous carbon matrix can provide high reactivity and facilitate more efficient electronic diffusion. For example, Ji et al. fabricated *α*‐Fe_2_O_3_ nanoparticle‐loaded carbon nanofibers by electrospinning and the subsequent thermal treatments, which provide a large surface area to enhance the contact between the electrolyte and electrode and delivers continuous conduction pathways for the transmission of lithium ions and electrons.^[^ ^40^ ^]^ However, since there is insufficient void space around *α*‐Fe_2_O_3_ nanoparticle to buffer the volume change, the long‐term cycling performance is not satisfactory. In order to preserve the integrity of the electrode upon cycling, a “bubble‐nanorod” structure was proposed, in which nanosized hollow Fe_2_O_3_ spheres uniformly dispersed in CF matrix (**Figure** **11**a,b).^[^ ^100^ ^]^ The capacity retentions of bubble‐nanorod‐structured Fe_2_O_3_–C composite nanofibers was 84% at 1 A g^−1^ after 300 cycles, while the hollow bare Fe_2_O_3_ nanofibers show rapid capacity decay owing to the continuous formation of SEI film (Figure 11c). The superior cycling stability of the nanofiber was attributed to the hollow nanospheres along with conductive carbon matrix could accommodate the volume change that occurs during cycling. Although metal oxides exhibit superior cycling stability, the relatively low conductivity limits their rate performance, especially at high current densities. To reduce charge‐transfer resistance of electrode, highly conductive metals are usually introduced into the metal oxide/carbon fiber system to improve the electronic conductivity of the composite fiber, thereby promoting rapid ion and electron transport.^[^ ^101^, ^102^ ^]^ This well‐designed hierarchical structure exhibited enhanced cycling stability even at very high current densities.

Alloying‐type materials (Si, Sn, Sb) have received widespread attention due to their ultrahigh discharge capacities. However, alloying‐type anodes suffer from the huge volume changes (up to 300%) during alloying and dealloying processes, resulting in electrode pulverization and separation from the current collector, further causing a rapid capacity fading.^[^ ^103^, ^104^, ^105^ ^]^ In this regard, combining 1D carbon fibers with alloying‐type materials can not only provide a buffer matrix to alleviate the volume change, but also promote the facile diffusion of lithium ion and improve electrode conductivity. Among alloying‐type materials, Si has received distinctive attention because of its high theoretical capacity up to 4200 mA h g^−1^ and natural abundance.^[^ ^106^ ^]^ Early in 2012, Gu and co‐workers probed the lithiation behavior of silicon nanoparticles attached to (embedded in) a CNF using in situ TEM and theoretical analysis.^[^ ^107^ ^]^ As shown in Figure 11d,e, compared to the attached structure, the lithiated silicon nanoparticles embedded in a carbon matrix will result in a high stress field, which may lead to the fracture of the CNF. Therefore, a proper spatial correlation of the silicon nanoparticle with the CF matrix is of great significance. An ideal configuration is to confine silicon nanoparticles in a hollow shell or a deformable coating material to allow the free expansion of silicon, thereby maintaining the structural integrity of the carbon matrix upon lithiation. For example, Zhu et al. reported a 3D flexible silicon/graphene/carbon OIHFs (3D FSiGCNFs) via ALD and subsequent electrospinning processes.^[^ ^65^ ^]^ The 3D hybrid structure with built‐in void space provided highly efficient channels for the fast electron transfer and allows silicon to expand freely without rupturing the entire electrode structure (Figure 11f). As a result, the 3D FSiGCNFs anode exhibited a remarkable cycling stability up to 500 cycles (Figure 11g). Instead of this complicated template coating and etching process, the Wang group directly used SiO*_x_* coating on Si NPs as the sacrificial template to constructed Si@void@C nanofibers.^[^ ^108^ ^]^ In this structure, the 3D CF matrix provides a high electrode–electrolyte contact area and fast lithium‐ion diffusion, and the void space between silicon and the carbon shell acts as a buffer to accommodate the huge volume variation. Compared to bare carbon nanofibers, the charge capacity of Si@void@C NFs has been significantly improved. In particular, coaxial electrospinning has been demonstrated with unique advantages for constructing core–shell fibers with well‐defined internal space. The Dou group proposed a core–shell fibrous composite in which the Si nanoparticles as the core and the mesoporous rutile TiO_2‐_ *_x_*/C nanocomposite as the shell.^[^ ^109^ ^]^ The void space in the core region could accommodate volume change of Si nanoparticles, while the oxygen‐deficient titania shell provides high conductivity and helps to maintain the structural stability.

Another alloying‐type anode material antimony (Sb) has also attracted wide interest because of its high theoretical gravimetric capacity of 660 mA h g^−1^.^[^ ^110^ ^]^ In order to alleviate the huge volume variation during lithium insertion/extraction process, numerous Sb/C fibrous structures have been designed to maintain the structural integrity. To buffer the volume change during Li–Sb alloying/dealloying processes, Wang et al. proposed “silica reinforcement” concept, encapsulating both Sb and silica nanoparticles into CNFs (SiO_2_/Sb@CNFs).^[^ ^111^ ^]^ Silica fillers not only provide additional lithium storage capacity but also reinforce the overall fibrous structure. The Sb nanoparticles are encapsulated into the fiber matrices, which efficiently buffer the volume changes during the repeated cycles. The obtained SiO_2_/Sb@CNF electrode demonstrates enhanced cycling performance at different current densities, indicating that silica fillers could greatly enhance the structural stability of the electrode material.

In summary, the homogeneous structure could shorten the ion diffusion length and offer oriented electronic/ionic transport pathway, however, there is not enough void space to accommodate the volume change of the active components. Therefore, constructing porous fiber matrix or rationally designing the void space around the active component can effectively limit structure degradation during cycling. The key to design of this type of fiber structure is to reasonably balance the relationship between void space and energy density, and to ensure the mechanical strength of the fiber framework. For OIHFs with functional interfaces, the strong chemical bond between active components with fiber matrix is essential for maintaining structural stability of composite electrode. In addition, the uniform elastic coating on the surface of active materials can prevent the active material from degrading and reduce side reactions.

#### 4.1.2 Sodium‐Ion Batteries

SIBs have demonstrated great potential for next‐generation energy storage devices due to low cost and abundant sodium resources.^[^ ^112^, ^113^, ^114^, ^115^ ^]^ Similar to LIBs, the SIBs operate through a “rocking‐chair” mechanism, in which the sodium ions shuttle between cathode and anode. SIBs have a typically carbonaceous anode and transition metal oxide cathode, meaning OIHFs can be designed to function at either electrode. The capacity of the anodes is severely limited by low power and energy densities due to the large size of Na^+^ and the less negative redox potential of Na^+^/Na, therefore, the development of suitable anode materials is more challenging in the research of SIBs.^[^ ^116^, ^117^, ^118^, ^119^ ^]^ Numerous inorganic materials (such as intercalation‐type, conversion‐type, and alloying‐type materials) have been proposed as anodes for SIBs by virtue of high theoretical capacity and low operating potential. In recent years, extensive research has been carried out to combine these inorganic materials with organic fibers. The unique 1D fibrous structure enables orientated electronic transport and promotes the accessibility between the active materials and the electrolyte, contributing to the satisfactory electrochemical performance of SIBs.

Carbon material is considered to be one of the most commonly used intercalation‐type material for SIBs. For example, wood cellulose fibers derived hard carbons have been developed as anodes for SIBs.^[^ ^120^ ^]^ Although this carbon fiber exhibits an excellent cycling stability, its specific capacity is far from satisfactory. Heteroatom doping provides more active sites for sodium storage, but the improvement in capacity is very limited.^[^ ^121^ ^]^ Apart from that, anatase TiO_2_ has been proved to be an ideal insertion‐type anode with relatively high reversible capacity and long cycle life. It has been proved that the completely chemical and structural reversibility of the insertion of Na^+^ into the TiO_2_ host.^[^ ^122^ ^]^

Metal oxide has been considered as promising conversion‐type anodes for SIBs. The main challenge of metal oxides in practical applications is the poor cyclic performance caused by huge volume changes during Na^+^ insertion and extraction process. Encapsulation of inorganic nanoparticles into carbon fibers is an effective method to suppress the volume expansion and accelerate the solid‐state ion diffusion. For example, Dirican et al. prepared amorphous carbon‐coated SnO_2_‐electrodeposited porous carbon nanofiber (PCNF@SnO_2_@C) composites, which possess higher capacity and capacity retention than bare SnO_2_ nanoparticles and porous carbon nanofibers.^[^ ^123^ ^]^ However, although this strategy suppresses the volume expansion to some extent, the nanostructured SnO_2_ inevitably tends to break during the cycling process. Defects (such as oxygen vacancies) can effectively regulate the electronic state of SnO_2_, thereby improving the cycling performance of the electrode. Accordingly, Ma et al. reported a robust SnO_2‐_ *_x_*/C nanoparticle‐impregnated carbon nanofibers, which is composed of highly uniform nanofibers with many macroporous holes embedded.^[^ ^66^ ^]^ The SnO_2‐_ *_x_*/C electrode shows excellent cyclic stability at a high current density (1 A g^−1^), with the discharge capacity of 565 mA h g^−1^ was retained after 2000 cycles. Over the past years, many metal oxides/carbon fiber composite structures such as CoO*_x_*/NC,^[^ ^124^ ^]^ *γ*‐Fe_2_O_3_/PCF,^[^ ^125^ ^]^ and HCF‐V_2_O_5_^[^ ^126^ ^]^ have also been widely utilized as anode materials for SIBs.

**Figure 12:**
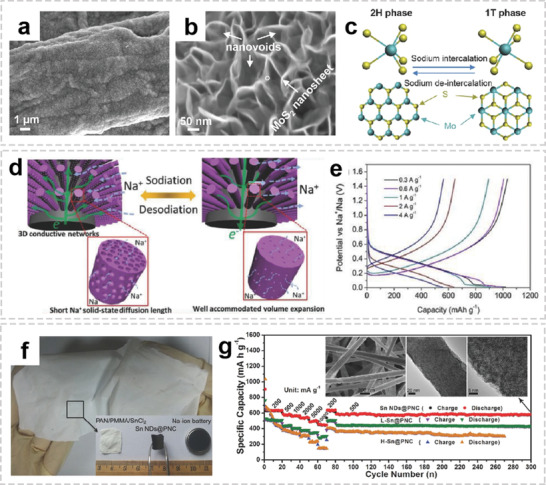
OIHFs for sodium‐ion batteries. a,b) SEM images of the as‐prepared freestanding MoS_2_@C. c) Schematic of the phase transition between 2H‐MoS_2_ and 1T‐MoS_2_ during sodium intercalation/deintercalation. a‐c) Reproduced with permission.^[^ ^129^ ^]^ Copyright 2015, Wiley‐VCH. d) Schematic illustration of the electron/ion diffusion, volume variation of the mesoporous CoP_4_ arrays, and electron diffusion pathways on CF during the sodiation/desodiation. e) Charge/discharge profiles of CoP_4_/CF. d,e) Reproduced with permission.^[^ ^132^ ^]^ Copyright 2018, Wiley‐VCH. f) Digital photos of the PAN/PMMA/SnCl_2_ fiber membrane and the calcinated self‐supported Sn NDs@PNC electrode for Na‐ion batteries. g) Rate capability and cycling performance of Sn NDs@PNC, lower Sn content (L‐Sn@PNC), and higher Sn content (H‐Sn@PNC) electrodes, inset: SEM, TEM, and HRTEM images of Sn NDs@PNC after 300 cycles. f,g) Reproduced with permission.^[^ ^134^ ^]^ Copyright 2015, Wiley‐VCH.

Compared to metal oxides, metal chalcogenides possess the high electrical conductivity and weak M—S bonds, which are favorable for conversion reactions. Among them, MoS_2_, a 2D layered material with large interlayer distance, enables efficient Na^+^ storage via “intercalation–conversion” process.^[^ ^127^, ^128^ ^]^ Xie et al. reported a freestanding MoS_2_@C paper electrodes synthesized through hydrothermal process and subsequent calcination.^[^ ^129^ ^]^ The spatially dispersed MoS_2_ nanosheets construct nanovoids on carbon scaffolds, forming a 3D interconnected hierarchical structure (**Figure** **12**a,b). As shown in Figure 12c, during sodium intercalation process, the thermodynamically stable 2H‐MoS_2_ is converted to 1T‐Na*_y_* MoS_2_ due to the lattice strain induced by the Na^+^ intercalation process. The reverse transition can occur easily during deintercalation, with conversion to 2H‐MoS_2_ by a glide of the intralayer atomic planes. Nickel sulfide/fibers composites have also received much attention as a potential anode for SIBs due to high theoretical capacity of nickel sulfide (873 mA h g^−1^) based on the four‐electron conversion reaction (NiS_2_ + 4Na^+^ + 4e^−^ ↔ Ni + 2Na_2_S).^[^ ^130^ ^]^ In addition, metal phosphides have also been regarded as a promising anode for SIBs due to their high theoretical capacity and appropriate operating potentials.^[^ ^131^ ^]^ Sun et al. proposed a new strategy to grow a series of mesoporous metal phosphide nanoarrays (FeP_4_, CoP_4_, and NiP*_x_*) on carbon felt by a simple hydrothermal process combined with subsequent in situ phosphorization reaction.^[^ ^132^ ^]^ Due to the synergistic effect of the 3D conductive network and ultrasmall particle size, the resultant integrated electrodes demonstrate ultrahigh cycling stability as well as rate capability (Figure 12d,e).

Alloying‐type anodes (e.g., Ge, Sn, P, Sb) usually provide much higher gravimetric and volumetric specific capacities.^[^ ^133^ ^]^ In order to alleviate the volume change during Na^+^ insertion/extraction processes, a wide range of 1D nanostructured materials have been proposed to maintain the stability of the morphology. Remarkably, the Chen group reported the fabrication of Sn nanodots finely encapsulated in porous N‐doped carbon (Sn NDs@PNC) nanofibers via electrospinning and subsequent thermal treatment.^[^ ^134^ ^]^ The obtained Sn NDs@PNC fiber membrane was cut into self‐supported electrodes and directly assembled into SIBs (Figure 12f). Even after 300 cycles, the Sn NDs@PNC electrode shows high reversible capacity, further demonstrating that the tailored composite could restrain the pulverization and aggregation of Sn NDs (Figure 12g). Besides, P is another promising alloying‐type anode with the theoretical specific capacity of 2595 mA h g^−1^, P/carbon fiber composites have been proposed to facilitate the electrons diffusion kinetics and buffers the large volume change.^[^ ^135^ ^]^ However, tremendous volume expansion during the sodiation/desodiation process leads to pulverization of active materials, giving rise to the severe capacity loss. Therefore, Ma et al. introduced a graphene oxide coating on P/carbon fibers to improve the cycling stability of the composite anode.^[^ ^136^ ^]^ The surface graphene coating could effectively buffer huge volume change and promote electron transport. Furthermore, with a high theoretical capacity of 660 mA h g^−1^, antimony (Sb) has also been widely regarded promising anode candidate for SIBs. For example, Zhao et al. synthesized porous nitrogen‐doped C@Sb nanofibers through electrospinning and subsequent thermal treatment.^[^ ^137^ ^]^ The synergetic effects between the well‐dispersed ultrafine Sb nanoparticles and the N‐rich 3D conductive carbon network results in an ultrahigh rate capability of C@Sb anode.

Due to the larger ionic radius of Na^+^ (1.02 Å) than Li^+^ (0.76 Å), the electrode of SIBs generally suffers more seriously structural degradation than LIBs. When the active material is coated on the fiber surface, the large strain caused by volume change will cause the delamination of active material and subsequent poor long‐term cycling stability. Therefore, the microstructure of active materials should be rationally designed to overcome the disadvantages of huge volume change and sluggish electron transfer. In addition, encapsulation of ultrasmall nanoparticles into porous fiber could not only alleviate the volume change but also facilitate fast charge transfer through the highly conductive carbon framework. Whereas, the internal space between active material and fiber matrix should be carefully optimized to realize the balance of volumetric energy density and cycling stability.

#### 4.1.3 Lithium–Sulfur Batteries

The Li–S battery has attracted ever increasing attention for energy storage device due to the ultrahigh theoretical specific capacity (1675 mA h g^−1^) based on the electrochemical reaction of 16Li + S_8_ → 8Li_2_S.^[^ ^138^, ^139^ ^]^ The battery functions with a lithium‐based anode and a sulfur‐based cathode, with OIHFs primarily studied as sulfur hosts for cathode materials. The practical application of Li–S batteries is still obstructed by several drawbacks. The insulating nature of sulfur and sulfides limits the rate capabilities of these cathodes, and the adverse “shuttle effect” caused by the dissolution of polysulfides (Li_2_S*_x_*, 4 ≤ *x* ≤ 8) will result in low capacity and poor energy efficiency.^[^ ^140^, ^141^ ^]^ Significant research has been devoted to overcoming these issues by combining sulfur with carbonaceous fibers to increase the electrical conductivity as well as specific surface area.^[^ ^142^, ^143^ ^]^ Nevertheless, the interactions between the nonpolar carbon matrix and polar polysulfides are weak, leading to the poor cycling stability of the sulfur cathode. Therefore, combining the carbon fibers with the polar metal compounds would strongly anchor the polysulfides and effectively alleviate the shuttle effect.^[^ ^144^, ^145^, ^146^ ^]^

For example, the Hu group developed a 5 nm Al_2_O_3_‐coated carbonized mesoporous wood fiber (f‐CMWF) as a host to accommodate sulfur.^[^ ^147^ ^]^ The thin Al_2_O_3_ deposition layer could reduce the pore size of the electrically conductive CMWF (3 to 12 nm) to entrap small sulfur molecules and maintain the unique mesoporous structure of wood fiber. The obtained composite cathode delivers slow capacity decay rate of 0.046% per cycle at 400 mA g^−1^, while S/CMWF electrode suffers from fast capacity fading caused by the dissolution of sulfur on CMWF surface.

**Figure 13:**
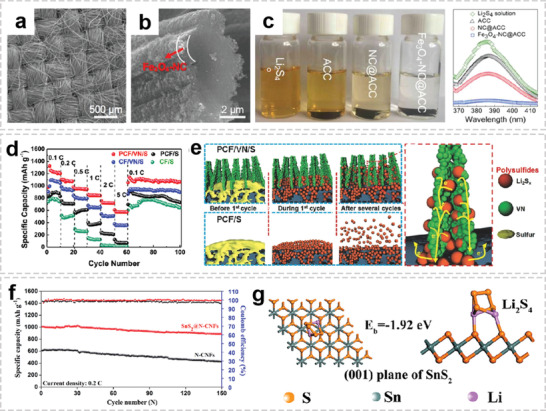
OIHFs for lithium–sulfur batteries. a) SEM image of carbon cloth. b) SEM image of pyrolyzed Fe_3_O_4_‐NC@carbon fiber. c) The picture and UV–vis adsorption spectra of the solutions after immersion. These clothes were immersed in 5 mm Li_2_S_4_ solution and the picture was taken after 30 min. a‐c) Reproduced with permission.^[^ ^148^ ^]^ Copyright 2018, Wiley‐VCH. d) Rate capability of four electrodes. e) Schematic illustration of dual blocking effects associated with “physical block and chemical absorption” for polysulfides in the PCF/VN/S electrode. d,e) Reproduced with permission.^[^ ^150^ ^]^ Copyright 2018, Wiley‐VCH. f) Cycling performance of N‐CNFs/Li_2_S_6_ and Co, N‐CNFs/Li_2_S_6_ electrodes with sulfur loading of 7.11 mg at 0.2 C. g) Top and side views of binding geometries and energies between (001) plane of SnS_2_ nanoplates and a Li_2_S_4_ molecule on DFT calculation. f,g) Reproduced with permission.^[^ ^154^ ^]^ Copyright 2020, American Chemical Society.

It has been proved that the combination of heteroatom doped carbon (N, P, B, etc.) and metal oxide could effectively enhance sulfur utilization. Lu et al. proposed the synthesis procedure of flexible porous carbon cloth coated with strongly coupled Fe_3_O_4_ and N‐doped carbon (**Figure** **13**a,b).^[^ ^148^ ^]^ It delivered a BET specific surface area of 265 m^2^ g^−1^ and showed a type IV adsorption–desorption isotherm, indicating the presence of both micropores and mesopores. After immersing the prepared cathode cloth in a Li_2_S_4_ solution, the solution become colorless within only 30 min without stirring, indicating the outstanding LPS adsorption capability (Figure 13c). They also found that the capacity of sulfur cathodes is strongly dependence on the amount of Fe_3_O_4_‐NC, indicationg that the synergy of Fe_3_O_4_ and N‐doped allowed efficient conversion between Li_2_S_4_ and Li_2_S.

Although these improvements have been achieved, many metal oxides are insulators or semiconductors, which are not conducive to the fast polysulfide conversion and lead to a low sulfur utilization. With high conductivity and strong affinity to lithium, metallic nitrides modified carbon fibers have been considered as an ideal anchoring material for polysulfides in Li–S battery. The Huang group designed biomimetic root‐like carbon/titanium nitride (TiN/C) mesoporous nanofibers, which were directly used as a freestanding cathode for high sulfur loading Li–S battery.^[^ ^149^ ^]^ The TiN/C@S cathode has a sulfur loading of 10.5 mg cm^−2^ and delivers a high reversible capacity of 790 mA h g^−1^ at 0.2 C. The 3D interconnected root‐like TiN/C matrix facilitates the high‐efficiency electron transfer, and the TiN could anchor the polysulfides via the polar Ti—S and N—S bond as well as accelerate the redox reaction due to its high catalytic effect. The Li_2_S precipitation experiments further confirmed that the capacity of Li_2_S precipitation on the TiN/C surface is much higher than that of the HCNF system. Zhong et al. fabricated a porous carbon fibers/vanadium nitride arrays (PCF/VN) composite scaffold, in which the VN nanobelts consist of 5–10 nm porous interconnected secondary nanoparticles.^[^ ^150^ ^]^ When applied as Li–S cathode, the PCF/VN/S electrode exhibited a superior rate performance (Figure 13d). The enhanced cycling performance can be explained by the schematic diagram in Figure 13e. The porous VN nanobelt arrays could strongly absorbed the polysulfides on the surface because of the polar–polar interaction, thus efficiently retarding the shuttle effect of soluble lithium polysulfides. Based on the above researches, the TiN‐VN heterostructures are used to composite with carbon fibers to improve the electrochemical performance of batteries.^[^ ^151^ ^]^ The cathode combines the merits of good conductivity and high surface polarization, thereby significantly suppressing the LiPS shuttle effect and achieving uniform Li deposition. Apart from that, the TiN‐VN heterostructures could effectively reduce the nucleation overpotential of Li_2_S and accelerate the interface electronics transfer kinetics of Li_2_S precipitation.

Due to their strong affinity to sulfur‐containing species, metal sulfides have been extensively explored for Li–S batteries. Combining metal sulfides with 1D fibers will effectively enhance the electrochemical reaction kinetics and inhibit the lithium polysulfide shuttle effect. Ahn and co‐workers designed a FeS@SPAN (iron sulfide nanoparticles in a sulfurized polyacrylonitrile nanofiber matrix) synergistic hybrid cathode via electrospinning process followed by a single‐step heating procedure.^[^ ^152^ ^]^ In this architecture, the good absorptivity of FeS facilitates the adsorption of polysulfides, while the iron nanoparticles formed during the discharge process catalyzes the oxidation of Li_2_S and improves the charge‐transfer kinetics. Furthermore, ivy‐structured MoS_2_ nanoflakes@nitrogen doped carbon nanofibers have been reported by Xue et al.^[^ ^153^ ^]^ The MoS_2_ nanoflakes can be chemically bonded to lithium polysulfides to promote fast redox reaction kinetics. When used in Li–S cathode, with a high sulfur loading of 7.11 mg, the hierarchical composite cathode demonstrates excellent cycling performance with a capacity decay rate of 0.08% per cycle over 250 cycles at 0.2 C. By contrast, a rapid capacity fade of N‐CNFs cathode was observed after 250 cycles owing to the weak adsorption of sulfur‐species during electrochemical reactions. Recently, Yao et al. prepared a 3D SnS_2_@N‐CNFs as a cathode for Li–S battery by electrospinning process followed by a hydrothermal technique.^[^ ^154^ ^]^ The average pore size of SnS_2_@N‐CNFs is around 2.15 nm, which has the unique advantage of absorbing polysulfide ions (the long‐order polysulfide species is about 2 nm). The SnS_2_@N‐CNFs with high sulfur loading displayed 0.08% capacity decay per cycle over 150 cycles at 0.2 C (Figure 13f). As shown in Figure 13g, the calculated binding energy of SnS_2_ and Li_2_S_4_ is −1.92 eV, indicating the strong polar–polar interaction between SnS_2_ and Li_2_S_4_.

Pursuing porous materials with high surface area and tunable pore structure to achieve high areal sulfur loadings is essential for achieving high energy density Li–S cathode. Further, introducing polar inorganic materials to strongly anchor the sulfur species by chemical bonding enables greater stability of the Li–S cathode. In light of these pursuits, the use of OIHFs in the form of porous and interconnected conductive metal nitrides and sulfides decorated organic fibers is critical for the rational design of cathode materials with high sulfur utilization. It is critical to achieve uniform growth of inorganic materials on the surface of fiber matrix to enable homogeneous sulfur loading and subsequent homogeneous lithium deposition during electrochemical reactions.

### 4.2 Supercapacitor

Supercapacitors have attracted tremendous attention mainly due to their high power density, long lifespan, and fast charge/discharge rates.^[^ ^155^, ^156^ ^]^ Supercapacitors can be divided into electric double‐layer capacitors (EDLCs) and pseudocapacitors according to the charge storage mechanism.^[^ ^157^, ^158^ ^]^ Porous carbon materials with ultrahigh specific surface area have been extensively utilized as electrode materials for EDLCs. OIHFs are often used as active electrode materials for pseudocapacitors (positive electrode for asymmetric supercapacitors) since they can store charges through rapid and reversible redox reactions occur at the interfaces of the electrode.^[^ ^159^ ^]^ Both EDLCs and pseudocapacitors can be produced in a wide variety of form factors that inform potential device applications. Here, we study the role of OIHF‐based supercapacitors by these form factors and geometries.

#### 4.2.1 Sandwich‐Shaped Supercapacitors

Sandwich‐shaped supercapacitors consist of two flat electrodes, which are separated by liquid or gel electrolytes and an ion‐porous separator material.^[^ ^160^ ^]^ In this configuration, the active electrode material can be directly grown or deposited on conductive substrate, or the slurry of active material can be coated on a metal current collector. Pseudocapacitive transition metal oxides (TMOs) are considered the ideal materials for pseudocapacitors as they could deliver high energy density.^[^ ^161^ ^]^ The poor electrical conductivity, and subsequent low power density of TMOs limit their wide application as stand‐alone electrodes in supercapacitors. Incorporating TMOs into highly conductive carbon‐based scaffolds such as carbon fibers is considered to be an effective strategy to overcome this problem. These OIHFs possess the merits of a high electrochemically active surface area (from both the carbon fibers and the TMO), pseudocapacitance (from the TMO), and relatively low internal resistance (from the carbon fibers).

For example, manganese oxides are promising materials for supercapacitors because of their high capacitance, wide electrochemical potential window, and environmentally benignity nature. Ramadan et al. prepared 3D interconnected binder‐free MnO@C nanofibers electrode via a facile one‐step electrospinning method.^[^ ^162^ ^]^ The carbon matrix not only facilitates the electronic transfer, but also provides a robust support to prevent the aggregation of MnO nanoparticles. When examined as the positive electrode in asymmetric device, the MnO@C delivers very high energy density (35.5 Wh kg^−1^) as well as cycling stability (127% capacity increasing after 3000 cycles). Instead of encapsulating MnO nanoparticles in fiber matrix, Lu et al. reported the fabrication of freestanding PAA@MnO_2_/PPy core–shell nanofibers from electrospun PAA fibers loaded with Mn^2+^ ions.^[^ ^163^ ^]^ The composite fibers are flexible, the PPy shell is about 50 nm and small MnO_2_ particles inside the fiber. The CV curves of PAA@MnO_2_/PPy at different scan rates show a rectangular shape, indicating an excellent capacitive behavior. In this composite, the MnO_2_ nanoparticles deliver high capacity, while the PPy shell could prevent the loss of MnO_2_ during the charge/discharge process.

RuO_2_ has also been considered an ideal candidate for supercapacitor electrodes due to its high specific capacitance and stable electrochemical reversibility. Jeon et al. reported high‐performance supercapacitor electrode by directly growing RuO_2_ nanorods on electrospun carbon nanofibers.^[^ ^15^ ^]^ The RuO_2_‐CNF electrode shows a larger CV‐integrated area than the CNF electrode, which is mainly attributed to the large surface area and electroactive functional sites of RuO_2_ nanostructures. In fact, conventional sandwich‐shaped supercapacitors are stiff and cumbersome, making it difficult to provide a perfect match for the requirements of flexible electronics. In the past decade, supercapacitors fabricated in flexible form have become a research hotspot.

#### 4.2.2 Fiber‐Shaped Supercapacitors

Fiber‐shaped supercapacitors (FSCs) are receiving significant attention due to the integration of the lightweight, tiny volume, high power density, excellent cycle lifetime, and fast charge–discharge capability.^[^ ^164^, ^165^ ^]^ They can be woven into different shapes or integrated into breathable textiles to meet the urgent demands of modern flexible and wearable electronics.^[^ ^166^, ^167^ ^]^ Although various carbonaceous fibers, such as carbon fibers, graphene fibers, and carbon nanotube fibers have been adopted to construct FSCs, the relatively low specific capacitance remains unsatisfactory for practical applications.^[^ ^168^ ^]^ OIHFs take advantages of both carbonaceous fiber and inorganic component, and they are widely employed as the electrodes for FSCs. In this subsection, three subcategories of OIHF‐based FSCs are described, including parallel structure, twisted structure, and coaxial structure.

**Figure 14:**
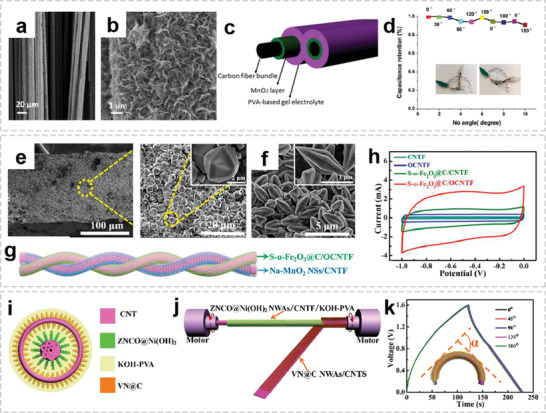
OIHFs for fiber‐shaped supercapacitors. a,b) FESEM images of carbon fiber@MnO_2_ nanosheets composite. c) 3D image of the assembled device. d) Capacitance retention at different angles. a‐d) Reproduced with permission.^[^ ^169^ ^]^ Copyright 2017, American Chemical Society. e) SEM image of MIL‐88‐Fe/OCNTF at increasing magnification. f) SEM image of S‐*α*‐Fe_2_O_3_@C/OCTNF electrode. g) Schematic illustration of our FASC device. h) Comparison of CV curves of pristine CNTF, OCNTF, S‐*α*‐Fe_2_O_3_@C/CTNF, and S‐*α*‐Fe_2_O_3_@C/OCTNF electrodes measured at a scan rate of 25 mV s^−1^. e‐h) Reproduced with permission.^[^ ^172^ ^]^ Copyright 2018, American Chemical Society. i) Cross‐sectional structure of the CFASCs. j) Wrapping of the VN@C NWAs/CNTS around the surface of the ZNCO@Ni(OH)_2_ NWAs/CNTF/KOH‐PVA. k) GCD curves of the as‐assembled CFASCs bent at various angles at a current density of 10 mA cm^−2^. i‐k) Reproduced with permission.^[^ ^175^ ^]^ Copyright 2017, American Chemical Society.

Parallel structure FSCs are fabricated by putting two electrodes parallel to each other, which is separated by gel electrolytes and supported by planar substrate. Rafique et al. fabricated a novel electrode structure in which a highly uniform porous MnO_2_ film was deposited on carbon fiber (**Figure** **14**a,b).^[^ ^169^ ^]^ The wearable device is assembled in a parallel structure, in which the gel electrolyte acting both as separator and ion mediator (Figure 14c). The capacitance retention at different flexing angles is shown in Figure 14d, and the almost 100% capacitance retention demonstrates the satisfactory bending stability of the obtained composite electrodes. In addition to the deposition technique, wet‐spinning is also widely explored for the fabrication of FSC electrodes. For example, Zhang et al. synthesized a Ti_3_C_2_T*_x_* MXene/PEDOT: PSS hybrid fiber through an easily scalable one‐step wet‐spinning approach.^[^ ^170^ ^]^ The PEDOT:PSS binder imparted spinnability to the nonspinnable Ti_3_C_2_T*_x_* MXene sheets and facilitated the intersheet charge transport. When repeatedly stretched to 100% strain and released, the elastic FSC shows 96% capacitance retention (361.4 F cm^−3^ in the unstretched state), which is attributed to the excellent strength and flexibility of the OIHFs.

FSCs with a twisted structure is constructed by two fiber electrodes twisted together, which are separated with a separator or filled with polymer gel electrolyte. The twisted electrodes show a significantly improved interface contact area compared with parallel electrodes, which is beneficial for electrochemical properties. For example, Peng group reported a fiber‐shaped asymmetric supercapacitor (AFSC) with the MnO_2_/PEDOT:PSS/CNT as positive electrode and OMC/CNT as negative electrode.^[^ ^171^ ^]^ The hybrid positive electrode was formed by growing MnO_2_ nanosheets onto a conducting polymer‐coated carbon nanotube fiber, which delivers high specific volumetric capacitance than PEDOT:PSS/CNT fiber. Further, in order to increase electrical conductivity and specific surface area, Zhou et al. fabricated specific MOF derived S‐*α*‐Fe_2_O_3_@C/OCTNF electrode via a facile hydrothermal method (Figure 14e,f).^[^ ^172^ ^]^ Then the flexible solid‐state twisted FSCs device was assembled by matching the obtained positive electrode with Na‐MnO_2_ NSs/CNTF negative electrode (Figure 14g). As presented in Figure 13h, the CV curve area of the S‐*α*‐Fe_2_O_3_@C/CNTFs is much higher than pristine CNTF electrode suggesting that the pseudocapacitive material S‐*α*‐Fe_2_O_3_@C makes higher contribution to the capacitance. Recently, the Zhang group designed a novel all‐solid‐state AFSCs with a maximum operating voltage of 3.5 V by adopting MnO*_x_* @TiN nanowires@carbon nanotube (NWs@CNT) fiber as the positive electrode and C@TiN NWs@CNT fiber as the negative electrode.^[^ ^173^ ^]^ For MnO*_x_* @TiN NWs@CNT fiber composite, the TiN NWs are uniformly wrapped by numerous MnO*_x_* nanosheets to form MnO*_x_* @TiN core–shell nanocomposites. Owing to the well‐designed hierarchical core/shell nanostructure, the obtained AFSCs delivers high power density (10.1 W cm^−3^) as well as excellent flexibility (92.7% capacitance retention after being bent for 1000 cycles at 90°).

FSCs with a coaxial structure consist of a core fiber electrode and an outer layer electrode, which are separated by a coaxial polymer gel. This aligned structure is conducive to the rapid charge transport and diffusion of electrolyte ions. All carbon coaxial FSCs were fabricated using a CMF bundle coated with multiwalled carbon nanotubes (MWCNTs) as a core electrode and a CNF film as an outer electrode.^[^ ^174^ ^]^ This electrode shows excellent flexibility, however, their low specific capacitance originating from the EDLC restrict the enhancement of their energy density. The Zhang group constructed a coaxial AFSCs by adopting zinc–nickel–cobalt oxide (ZNCO)@Ni(OH)_2_ nanowire arrays (NWAs)/carbon nanotube fiber (CNTF) as the core electrode, a VN@C NWAs/carbon nanotube strip (CNTS) as the outer electrode (Figure 13i,j).^[^ ^175^ ^]^ From the GCD curves shown in Figure 14k, there were negligible changes with different bending angles from 0° to 180°, indicating the exceptional flexibility of the CFASC. The improved performance is thought to be caused by the enhanced electrical conductivity of the core electrode and the increased direct contact area of the coaxial structure. Afterward, this group further designed a novel all‐solid‐state coaxial asymmetric FSC by using Au‐nanoparticle‐doped‐MnO*_x_* @CoNi‐alloy@carbon nanotube (Au–MnO*_x_* @CoNi@CNT) as the core electrode.^[^ ^176^ ^]^ The coaxial FSC exhibited a high volumetric energy density (0.53 mWh cm^−3^) as well as a superior cycle stability (90% retention after 10 000 cycles) owing to the high ion accessible surface area.

Compared with conventional sandwich‐shaped supercapacitors, FSCs possess great flexibility and can bear deformations in almost all dimensions. For device configurations, because of the limited contact area between the electrode and the electrolyte, the energy density of parallel structure is greatly limited. The current trend in FSCs is the development of twisted and coaxial structure with improved interface area. In terms of structural design, constructing fiber matrix with high surface area to increase the loading of active components could effectively increase energy density and capacitance.

### 4.3 Electrocatalysis

Electrocatalytic reactions including the oxygen reduction reaction (ORR), oxygen evolution reaction (OER), and hydrogen evolution reaction (HER), are at the heart of emerging green and renewable electrochemical energy technologies. Highly efficient electrocatalysts with low required overpotentials are needed to push the development of these clean energy technologies. Instead of choosing highly efficient catalysts, support materials also play an important role in stabilizing nanoparticles to fully expose active sites. Carbon fibers show great advantages including fast electron transfer and preferential exposure of reactive sites, which have been widely utilized as conductive support to load active catalyst. Therefore, OIHFs with large specific surface areas, abundant accessible active sites, as well as fast electron transport (provided by the carbon fibers) have received great attention as active electrocatalysts. Some representative electrocatalysts and corresponding electrochemical performance are listed in **Table** **2**.

**Table 2:** Performance comparison of different OIHFs electrode for ORR/OER/HER applications

Application	Materials	Structure	Synthesis technique	Electrolyte	*E*_onset_ ^a)^ [mV]	*η*_10_ ^b)^ [mV)]	*E*_1/2_ ^c)^ [mV]	Tafel plot [mV dec^−1^]	Ref.
ORR	N‐Co_3_O_4_/CC	Funct‐	Hydrothermal	0.1 m KOH	940	–	–	57.6	^[^ ^183^ ^]^
	Co_3_O_4‐_ *_x_* HoNPs@HPNCS	Funct‐	Impregnation	0.1 m KOH	–	–	834	–	^[^ ^184^ ^]^
	FeNC–S–Fe*_x_* C/Fe	Funct‐	Interfacial growth	0.1 m HClO_4_	–	–	821	71	^[^ ^85^ ^]^
	Co/Co–N–C	Funct‐	ED	0.1 m KOH	820	–	–	–	^[^ ^189^ ^]^
	NiCo@N–C NFs	Homo‐	Electrospinning	0.1 m KOH	–	–	–	65	^[^ ^185^ ^]^
	OM‐NCNF‐FeN*_x_*	Homo‐	Electrospinning	0.1 m KOH	850	–	774	63	^[^ ^187^ ^]^
	Co–N–PCNFs	Homo‐	Electrospinning	0.5 m H_2_SO_4_	950	–	810	–	^[^ ^188^ ^]^
OER	CP@Ni–P NS	Funct‐	ED	1 m KOH	–	–	–	73	^[^ ^196^ ^]^
	MnO_2_/CFP	Funct‐	ED	1 m KOH	–	390	–	104.4	^[^ ^22^ ^]^
	MoO_3_/Ni–NiO/CC	Funct‐	ED	1 m KOH	–	62	–	60	^[^ ^199^ ^]^
	Ni–Co/CF	Funct‐	Hydrothermal	1 m KOH	–	302	–	43.6	^[^ ^89^ ^]^
	MCCF/NiMn‐MOFs	Funct‐	Hydrothermal	1 m KOH	–	280	–	86	^[^ ^200^ ^]^
	Ni|MnO/CNF	Homo‐	Electrospinning	1 m KOH	–	–	–	71.5	^[^ ^198^ ^]^
HER	NiO/CFP	Funct‐	Cation exchange	1 m KOH	–	110	–	100	^[^ ^203^ ^]^
	Ni, Zn–CoO/CFP	Funct‐	Cation exchange	1 m KOH	–	53	–	–	^[^ ^25^ ^]^
	CC@N–CoP	Funct‐	Hydrothermal	0.5 m H_2_SO_4_	–	42	–	41.2	^[^ ^207^ ^]^
	Mo_1.33_W_0.67_C@ NC NWs/CC	Funct‐	Hydrothermal	0.5 m H_2_SO_4_	39	115	–	58.5	^[^ ^214^ ^]^
	Ni_2_P@NPCNFs	Homo‐	Electrospinning	0.5 m H_2_SO_4_	–	63.2	–	35	^[^ ^56^ ^]^
	Ni_2‐_ *_x_* Co*_x_* P/NC NF	Homo‐	Electrospinning	1 m KOH	68	130	–	70	^[^ ^54^ ^]^
	Ni_3_Fe@N‐C NT/NFs	Homo‐	Electrospinning	1 m KOH	–	72	–	98	^[^ ^215^ ^]^

^a)^ *E*_onset_: onset potential

^b)^ *η*: overpotential

^c)^ *E*_1/2_: half‐wave potential.

#### 4.3.1 Oxygen Reduction Reaction

The ORR is the core reaction of numerous energy conversion and storage technologies, such as regenerative fuel cells, metal–air batteries, and water‐splitting systems.^[^ ^177^, ^178^, ^179^ ^]^ The direct 2‐electron or 4‐electron pathway are two possible ways for reducing oxygen, of which the 4‐electron pathway is more desirable for efficient ORR electrocatalysis. Pt‐based catalysts are regarded as the most efficient ORR catalysts, however, their rarity, high cost, as well as inferior durability severely impeded their large‐scale applications.^[^ ^180^, ^181^, ^182^ ^]^ Owing to these facts, exploring inexpensive and highly efficient nonprecious metal catalysts have emerged as a promising alternative to Pt catalysts.

**Figure 15:**
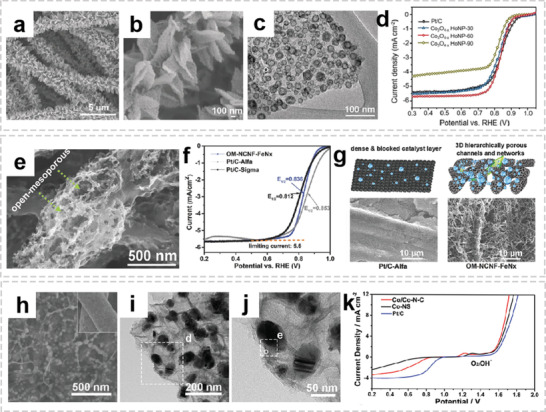
OIHFs for oxygen reduction reaction. a,b) SEM images of Co_3_O_4‐_ *_x_* HoNPs@HPNCS. c) TEM image of one carbon flake on the fibers. d) ORR polarization curves. a‐d) Reproduced with permission.^[^ ^184^ ^]^ Copyright 2019, Wiley‐VCH. e) High‐resolution SEM image of the fabricated OM‐NCNF‐FeN*_x_*. f) ORR polarization curves of OM‐NCNF‐FeN*_x_* and Pt/C catalysts in 0.1 m KOH. g) SEM images and schematic pictures of Pt/C ink (left) and OM‐NCNF‐FeN*_x_* ink (right) on the carbon cloths. e‐g) Reproduced with permission.^[^ ^187^ ^]^ Copyright 2018, Wiley‐VCH. h) SEM image of Co/Co–N–C. i,j) TEM images of Co/Co–N–C. k) Linear sweep voltammetry curves tested in O_2_‐saturated 0.1 m KOH electrolyte at the scan rate of 5 mV s^−1^. h‐j) Reproduced with permission.^[^ ^189^ ^]^ Copyright 2019, Wiley‐VCH.

TMOs possess the advantages of low‐cost and rich redox chemistry, however, the reaction activity of the TMOs‐based catalyst is far below the expectation owing to their relatively poor conductivity. It is known that by hybridizing TMOs with carbon fibers effectively enhances the electrical conductivity. The Yang group reported a controllable N‐doping strategy to regulate the electronic structures of Co_3_O_4_ nanowire arrays on carbon cloth.^[^ ^183^ ^]^ They confirmed that the conductivity of Co_3_O_4_ and the adsorption energy of O_2_ increases with the increase of N dopant, which is conductive to boosting the ORR activity. Furthermore, oxygen vacancies are considered an effective way to manipulate the electronic structures of TMOs. The Guo group reported a novel method to controllably construct oxygen‐vacancy‐rich Co_3_O_4_ hollow particles on a hierarchically porous carbon fiber (**Figure** **15**a–c).^[^ ^184^ ^]^ The abundant oxygen vacancies not only enhance the electrical conductivity of Co_3_O_4_ nanoparticles, but also regulate the electronic structures and the number of active sites. As a result, the ORR half‐wave potential of the obtained catalyst is 0.834 V (Figure 15d), exceeding that of state‐of‐the‐art Pt/C.

Bimetallic alloys have been applied as an efficient ORR electrocatalysts due to their superior catalytic activity in comparison to their individual entities. Similar to TMOs, bimetallic alloy catalysts are also facing the problem of poor conductivity. Due to the synergy of high conductivity and catalytic activity, bimetallic alloy functionalized hybrid fibers have been demonstrated great potential as active ORR catalysts. Fu et al. reported a novel hybrid catalyst, in which the NiCo alloy nanoparticles decorated on N‐doped carbon nanofibers by a facile electrospinning.^[^ ^185^ ^]^ Their superior catalytic activities than pure N‐doped carbon nanofibers can be attributed to the decreased charge transfer resistances and the formation of highly active hydroxylation of Ni/Co.

Recently, single‐atom catalysts have attracted widespread research interests owing to their preeminent catalytic activity and superior selectivity distinct from their nanosized counterparts.^[^ ^186^ ^]^ Transition metal‐based nitrogen‐doped carbons (M–N–C, M is Fe, Co, and Ni) have emerged as a promising alternative to Pt because of its desirable catalytic activity. Inspired by fibrous string structures of bufo‐spawn, Cheng et al. designed a novel atomic Fe–N*_x_* sites coupled open‐mesoporous N‐doped‐carbon nanofibers as advanced ORR catalyst (Figure 15e).^[^ ^187^ ^]^ Figure 15f shows the ORR polarization curve, the half‐wave potential of obtained catalyst is comparable to that of the commercial Pt/C catalyst. Their superior catalytic performance can be explained by Figure 15g, the interconnected mesoporous structure could fully expose the Fe–N*_x_* sites and improve the mass transfer performance, while a dense and blocked catalyst layer in Pt/C catalyst may limit the air diffusion efficiency. Of note, the iron‐carbide‐containing iron clusters are easily formed during the synthesis of atomically dispersed Fe–N–C catalysts, so it is difficult to maximize the reaction efficiency. Recent research has proved that the low electronegativity of S modifies the electronic structure of Fe–N active center, which is beneficial to further improve ORR activity. Qiao et al. utilized sulfuration to boost the ORR performance of an atomically dispersed Fe–N–C electrocatalyst.^[^ ^85^ ^]^ Owing to the synergistic effect of the FeN_4_ sites and Fe—S bond in the Fe–N–C carbon framework, the obtained FeNC–S–Fe*_x_* C/Fe composite exhibits superb ORR reactivity.

Co–N–C catalysts are also a promising candidate for the ORR. Recently, zeolitic‐imidazole frameworks (ZIFs) have been widely used as effective precursors to construct atomically dispersed Co–N–C sites or nitrogen‐doped carbon supported Co nanoparticles. For example, He et al. proposed a novel 3D fibrous Co–N–C catalyst through electrospinning followed by two‐step thermal treatments.^[^ ^188^ ^]^ The abundant ZIFs‐derived micropores effectively increased the utilization of single‐atom cobalt sites and promote the mass transfer of both the reactant and product. Nevertheless, there are usually limited amounts of Co–N–C catalytic sites derived from ZIF precursor, it is still challenging to construct the Co–N–C catalysts with sufficient catalytic active sites. In order to provide efficient ORR active sites, a novel hierarchical structure of Co nanoislands rooted on Co–N–C nanosheets supported by carbon felts was proposed.^[^ ^189^ ^]^ As illustrated in Figure 15h–j, the Co NPs with a diameter of 50 nm are homogeneously dispersed on the surface of nanosheets, forming a unique 3D hierarchical nanostructure. Owing to the high ratio of active sites and good conductivity, the Co/Co–N–C catalyst shows superior oxygen catalytic activity, which is comparable to those of the commercial Pt/C catalyst (Figure 15k).

#### 4.3.2 Oxygen Evolution Reaction

OER is the core step of anodic reaction, in which the H_2_O in the solvent is oxidized to O_2_.^[^ ^7^ ^]^ It requires a 4‐electron reaction pathway under high oxidative potential to release oxygen, so exploring highly efficient oxygen electrocatalysts to decrease the overpotential are urgently needed. At present, IrO_2_ and RuO_2_ are identified as the best OER catalysts because of their superior stabilities in acidic environments, but their scarcity and high cost impede the scale‐up of these materials for commercial applications.^[^ ^190^, ^191^ ^]^ To date, many efforts have been devoted to exploit nonprecious metal‐based catalysts, such as metal alloys, oxides, hydroxides, phosphates, and carbides.^[^ ^192^, ^193^, ^194^ ^]^ Due to the simplicity of functionalization, fibrous materials are considered to be an ideal substrate for growing the above functional materials.

**Figure 16:**
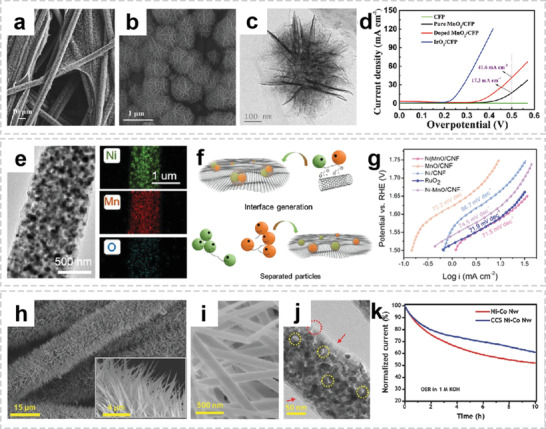
OIHFs for oxygen evolution reaction. a,b) FESEM and c) TEM images of the metal‐ion‐doped MnO_2_ ultrathin nanosheet/CFP composite electrode. d) LSV curves of the four electrodes measured in 1 m KOH with a scan rate of 5 mV s^−1^. a‐d) Reproduced with permission.^[^ ^22^ ^]^ Copyright 2017, Wiley‐VCH. e) TEM image and elemental mapping images of the Ni|MnO/CNF catalyst. f) Illustration of the CNT‐COOH anchoring strategy, which tends to initiate bimetallic heterointerfaces in the CNF. g) Tafel slopes of the prepared catalysts and RuO_2_. e‐g) Reproduced with permission.^[^ ^198^ ^]^ Copyright 2019, Wiley‐VCH. h) High‐magnification SEM images of CCS Ni–Co NWs on carbon fiber fabric. i) HRSEM and j) TEM images of CCS Ni–Co NWs. k) Chronoamperometric responses of catalytic electrodes at constant potentials for OER. h‐k) Reproduced with permission.^[^ ^89^ ^]^ Copyright 2016, Wiley‐VCH.

Recently, TMOs have been extensively investigated for OER catalyst owing to their low cost, natural abundance, and thermodynamic stability in oxidative environments. For example, Chen et al. developed a simple electrodeposition method to directly grow ultrathin Co_3_O_4_ layers on carbon fibers in the flexible carbon cloth (CC).^[^ ^195^ ^]^ The ultrathin Co_3_O_4_/CC electrode shows superior catalytic activity, which is attributed to the high utilization degree of active materials as well as rapid electron transport. However, the catalytic performance of pristine TMOs is generally hindered by poor conductivity and undesired adsorption energies (∆*E*) of the reaction intermediates. Several approaches have been proposed to improve the catalytic activity of TMOs‐based OIHFs, such as chemical doping, introducing oxygen vacancies, and fabricating heterostructures. Wang et al. fabricated a novel vertically aligned Ni–P nanosheets functionalized carbon fiber paper electrode via electrodeposition and phosphorization processes.^[^ ^196^ ^]^ During the OER catalysis in alkaline solution, the surface of Ni–P is transformed to NiO covered with a thin Ni(OH)*_x_* layer, forming a Ni–P/NiO/Ni(OH) *_x_* heterojunction, which plays a critical role in improving the electrocatalytic performance of OER. It has been confirmed that the doping of metal ions into TMOs could significantly improves the conductivity of the pristine material. Ye et al. fabricated metal‐ion (Fe, V, Co, and Ni)‐doped MnO_2_ ultrathin nanosheet/CFP composite electrodes through a facile anodic coelectrodeposition (**Figure** **16**a–c).^[^ ^22^ ^]^ During the electrodeposition process, Fe, V, Co, and Ni metal ions could replace the Mn ion in the regular octahedron [MnO_6_] units to form ultrathin nanosheet structures. The oxygen vacancies in MnO_2_ were easily formed due to their different valences between these metal ions and Mn^4+^, which is conductive to increase the number of the active sites for OER. When the OER overpotential was 500 mV, the obtained composite electrode achieved a high current density of 41.6 mA cm^−2^, while the bare CFP shows negligible catalytic current, indicating that the metal ions doping could significantly improve electrocatalytic performances (Figure 16d).

Metal/metal compounds heterostructures have unique features of abundant active sites, adjustable heterointerfaces, and fast interfacial electron transfer, which enables them as potentially promising bifunctional catalysts.^[^ ^197^ ^]^ Ji et al. reported the construction of Ni|MnO heterointerfaces in porous carbon nanofibers through a facile electrospinning‐calcination process (Figure 16e).^[^ ^198^ ^]^ Owing to the efficient anchoring interface of porous 1D CNF, the dissimilar metals tend to form strong bonding interfaces instead of two separated phases (Figure 16f). As shown in Figure 16g, Ni|MnO/CNF exhibited the smallest Tafel slope of 71.5 mV dec^−1^, which can be attributed to the Ni|MnO heterointerface efficiently decreases the mass and electron transfer barriers. Furthermore, the multicomponent system with different heterostructures could also reduce the energy barriers of HER and OER, respectively, thereby synergistically promoting the overall water splitting processes.^[^ ^199^ ^]^

Bimetallic catalysts have attracted special attention due to their synergistic effects of different metals. Bae et al. reported an integrated 3D architecture, in which the Ni–Co nanowires grown on a carbon fiber fabric with a seamless conductive carbon shell.^[^ ^89^ ^]^ As shown in Figure 16h–j, the mesoporous nanowires (NWs) with the smooth carbon shell are uniformly distributed on the carbon fiber. With the protection of the carbon coating, the core–shell Ni–Co NWs electrode retains 62% of its initial current density after 10 h, indicating an enhanced stability and durability for OER (Figure 16k). Lou group recently reported a facile hydrothermal process to directly grow NiMn layered double hydroxide (LDH) nanosheets on multichannel carbon fibers.^[^ ^200^ ^]^ Through a ligand exchange reaction, these NiMn‐LDH nanosheets are in situ transformed into NiMn‐MOF nanosheets. The NiMn‐MOFs nanosheets could completely expose the highly active bimetal centers on the surface, which helps increase the amounts of active sites for OER.

#### 4.3.3 Hydrogen Evolution Reaction

Hydrogen has gained attentions as one of the most appealing fuels owing to its high‐energy density and zero‐emission characteristics.^[^ ^201^ ^]^ The hydrogen evolution reaction is a classic two‐electron reaction that occurs at the cathode of an electrochemical water splitting device.^[^ ^202^ ^]^ Although Pt‐based catalysts are regarded as the best catalysts for the HER, their high cost and scarcity hinder large‐scale implementation. Therefore, intensive efforts have been made to develop nonprecious metal‐based catalysts to replace traditional Pt‐based catalysts.

**Figure 17:**
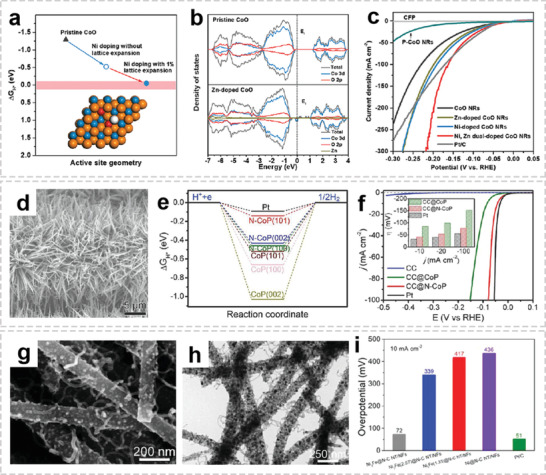
OIHFs for hydrogen evolution reaction. a) DFT calculated hydrogen adsorption free energy, Δ*G*_H∗_, on pristine CoO, Ni‐doped CoO (with ≈11% surface Ni dopant concentration), and the strained Ni‐doped CoO (with 1% tensile strain). b) Density of states of pristine CoO and Zn‐doped CoO (with ≈2% Zn dopant concentration). c) LSV of the four catalysts recorded in 1 m KOH. a‐c) Reproduced with permission.^[^ ^25^ ^]^ Copyright 2018, Wiley‐VCH. d) SEM image CC@N‐CoP. e) Free energy diagram for hydrogen (H*) adsorption at the Co–Co bridge site on several low‐index planes of CoP with and without N doping. f) LSV curves of the four catalysts (the inset compares overpotentials at varied current densities). d‐f) Reproduced with permission.^[^ ^207^ ^]^ Copyright 2018, Wiley‐VCH. g) SEM image and TEM images of the as‐synthesized Ni_3_Fe@N‐C NT/NFs. i) Overpotentials of the five catalysts at a current density of 10 mA cm^−2^. g‐i) Reproduced with permission.^[^ ^215^ ^]^ Copyright 2018, Wiley‐VCH.

TMOs are generally considered as HER inactive materials due to their inappropriate hydrogen adsorption energy as well as inherently poor electronic conductivity. Although coupling of TMOs with highly conductive carbon fibers could improve electron transport, the intrinsic electronic conductivity of active materials also needs to be improved. It was found that the formation of oxygen vacancies in TMOs could enhance the conduction of the catalysts. Qiao group used DFT computations to investigate the electronic band structure of TMOs with O‐vacancies. When O‐vacancy is introduced in NiO, some new electronic states appear near the Fermi level, leading to higher electronic conductivity of NiO.^[^ ^203^ ^]^ This group further confirmed that dual metal doping can precisely control the electronic structure of TMOs, thereby significantly improving the intrinsic and apparent activity of TMOs for alkaline HER (**Figure** **17**a–c).^[^ ^25^ ^]^

Transition metal phosphides (TMPs) are a promising class of nonprecious metal catalysts due to their hydrogenase‐like catalytic mechanism.^[^ ^204^, ^205^, ^206^ ^]^ Various TMPs such as FeP, CoP, Ni_2_P, and MoP have coupled with conductive carbon fiber for active HER catalysts. For example, the Bao group presented an electrospinning‐based reduction approach to in situ generate NiP nanoparticles in N‐doped porous carbon nanofibers (Ni_2_P@NPCNFs).^[^ ^56^ ^]^ This method could be applied to a series of pea‐like M*_x_* P@NPCNFs, including Fe_2_P@NPCNFs, Co_2_P@NPCNFs, and Cu_3_P@NPCNFs. The M*_x_* P@NPCNFs offer excellent catalytic ability and durability in both neutral and basic media, whereas the bare carbon cloth shows a nearly negligible HER performance. In addition to constructing a N‐doped carbon matrix, many studies have been devoted to optimizing the HER activity by turning the electronic structure of active TMPs. Zhou et al. reported a N‐doped CoP electrocatalyst for the HER (Figure 17d).^[^ ^207^ ^]^ They proved that N doping could modulate the electronic structure of orthorhombic CoP and the H adsorption on the CoP surface. As a result, the free energy of adsorbed H is closer to thermos‐neutral states, leading to the significantly improved catalytic activity of CoP (Figure 17e,f). Besides, it has been demonstrated that the composition of bimetallic phosphides can be easily tailored to fulfill electronic modulation on active‐sites.^[^ ^54^ ^]^

In addition, other transition‐metal based compounds, such as alloys,^[^ ^208^ ^]^ sulfides,^[^ ^209^ ^]^ selenides,^[^ ^210^ ^]^ nitrides,^[^ ^211^ ^]^ and carbides,^[^ ^212^ ^]^ have also demonstrated impressive HER activity. For instance, a facile topotactic transformation strategy has been proposed to grow a series molybdenum‐based compounds (MoX*_n_*, MoX*_n_* = MoP, MoS_2_, Mo_2_C, MoN, and MoO_2_) on carbon fiber paper.^[^ ^213^ ^]^ These electrodes present high HER activity and stability in pH‐universal media, especially, MoP‐based electrode shows comparable HER performance over platinum benchmark. Recently, the formation of a graphitic layer has been proposed to protect the transition metals from liquid electrolyte corrosion and enhance their conductivity. Li et al. developed a novel self‐supported electrode by growing nitrogen‐doped carbon encapsulated bimetallic molybdenum–tungsten carbide nanoparticles on carbon cloth.^[^ ^214^ ^]^ Owing to the protection of carbon shell, the Mo*_x_* W_2−_ *_x_* C nanoparticles maintain their structural stability during the electrochemical process. Further, the N doping effectively enhances the electron density in the carbon shell and provides extra nonmetallic active sites. The Tang group reported a facile electrospinning technique to encapsulate Ni_3_Fe nanoparticle in N‐doped carbon nanotube‐grafted carbon nanofibers.^[^ ^215^ ^]^ As shown in Figure 17g,h, most of the Ni_3_Fe nanoparticles are homogenously anchored on the fiber framework and carbon nanotubes are rooted from the fiber surface. Owing to the unique hierarchical nanoarchitecture, the obtained electrode exhibits an impressive HER performance with a low overpotential of 72 mV at a current density of 10 mA cm^−2^ (Figure 17i).

In summary, nonprecious transition metals including Fe, Co, Ni, and Mo, along with their alloys, oxides, nitrides, sulfides, and phosphides, have been considered as potential candidate for electrocatalysts. Their catalytic performance can be further improved by elements doping, introducing oxygen vacancy, and fabricating heterostructures. Coupling these above inorganic materials with heteroatom‐doped carbon fibers could significantly enhance the intrinsic electronic conductivity and electrocatalytic activity. For the structural design of OIHFs, constructing hierarchically porous 1D structure to anchor active materials could maximize electron transfer and the utilization of active sites. Moreover, loading the active material on the conductive carbon supports hides the catalytically inactive carbon surface and presents the inorganic active sites to the solution, significantly improving electrocatalytic performance. Increasing the inorganic/organic material ratio, by fabricating freestanding electrodes without resistive binders will enable the rational of components to achieve the optimal power‐to‐weight ratio for electrocatalysis.

## 5 Conclusions and Perspective

Significant achievements have been made in the development of functional OIHFs. This review has summarized the advances on OIHFs from their controllable structural design to electrochemical energy applications. Primarily, to promote the advanced structural design of OIHFs, an in‐depth understanding of the basic concepts such as composition, interfaces, and synthesis routes is needed. Further, we specially emphasize the fabrication approaches of OIHFs with homogeneous structures, multiple interiors, and functional interfaces. These well‐designed OIHFs will benefit electrochemical energy storage and conversion applications, such as batteries, supercapacitors, and electrocatalysts. Despite the significant progress has been made, there remain key challenges that need to be overcome to realize the full potentials of OIHFs.

Presently, electrospinning remains the most commonly used method for the fabrication of OIHFs. However, limitations exist for electrospinning, specifically with regard to achieve a controlled distribution of organic and inorganic components. Future research should be focused on precisely control over the proportion and spatial location organic/inorganic species at molecular level. Moreover, though a great many interface modification approaches have been developed to endow OIHFs with various interfacial properties, the interface interaction between the inorganic component and the fiber matrix is usually weak van der Waals force or electrostatic interaction. New synthetic strategies should be explored to couple inorganic species and organic fibers through strong interfacial interactions, such as covalent bonding.

In terms of structural engineering, modulating the electronic structure by elemental doping opens up a new paradigm to improve intrinsic reaction activity of OIHFs. Porous fibers with high specific surface areas and well‐defined porosity increase the diffusion rate and accelerate mass transport of reactants to the electrocatalyst, which is beneficial to enhancing the reaction efficiency in both energy storage and conversion systems. In fact, the structural engineering of OIHFs for various electrochemical energy applications is quite different. For electrode materials in rechargeable batteries, the rational void space between active material and fiber matrix is necessary for accommodating structural stress, including volumetric expansion and contraction, during electrochemical reactions. It is critical to coordinate the void space and volumetric energy density to achieve high capacity as well as long cycle life. As for electrocatalytic reactions, constructing electrode materials with high specific surface to fully expose the accessible active sites will be beneficial for fabricating optimal catalysts. Further, the interface assembly of highly dispersed ultrasmall nanocrystals and fiber frameworks is a desirable way to minimum side reactions and prevent the aggregation of active materials.

For electrochemical energy applications, significant effort is needed to develop facile and versatile methods to fabricate OIHFs in a cheap and scalable manner. In this context, it is crucial to construct freestanding electrode without additives to ensure good electrical contact between catalyst and support. Although OIHFs have been intensively studied, fiber‐shaped electronics remain an immature technology and are far from commercial applications. Exploring new approaches to fabricate integrated hybrid fibrous electrode with high mechanical and electrical properties are an essential future focus of research. OIHFs are diverse and powerful materials, and a deep understanding of component properties and the organic‐inorganic material interface will guide the selection and evolution of OIHFs for electrochemical energy devices.

## Conflict of Interest

The authors declare no conflict of interest.
